# ﻿Three new genera and one new species of leaf insect from *Melanesia* (Phasmatodea, Phylliidae)

**DOI:** 10.3897/zookeys.1110.80808

**Published:** 2022-07-05

**Authors:** Royce T. Cumming, Stéphane Le Tirant

**Affiliations:** 1 Montreal Insectarium, 4101 rue Sherbrooke est, Montréal, Québec, H1X 2B2, Canada Montreal Insectarium Montreal Canada; 2 Richard Gilder Graduate School, American Museum of Natural History, New York, NY 10024, USA Richard Gilder Graduate School, American Museum of Natural History New York United States of America; 3 Biology, Graduate Center, City University of New York, NY, USA City University of New York New York United States of America

**Keywords:** Aru Islands, camouflage, Indonesia, mimicry, new combination, Papua New Guinea, Phasmida, walking leaf

## Abstract

With the first large-scale Phylliidae molecular phylogeny recently published adding a great deal of clarity to phylliid diversity, several of the rarer species which could not be included were methodically and morphologically reviewed. This review resulted in identification of numerous substantial morphological features that suggest there are Melanesian clades that create polyphyletic groups within the phylliids which should instead be taxonomically recognized as unique. These rarer *Melanesia* species have historically been considered to be southern representatives of the *Pulchriphyllium* Griffini, 1898 sensu lato. However, there are notable morphological differences between the *Pulchriphyllium* sensu stricto and the “*schultzei*” group. Therefore, two new genera are erected, *Vaabonbonphyllium***gen. nov.** from the Solomon Islands and Papua New Guinea and *Rakaphyllium***gen. nov.** from New Guinea and the Aru Islands. Erection of these two new genera warrants the following new combinations: *Rakaphylliumschultzei* (Giglio-Tos, 1912), **comb. nov.**, *Rakaphylliumexsectum* (Zompro, 2001b), **comb. nov.**, and *Vaabonbonphylliumgroesseri* (Zompro, 1998), **comb. nov.** Additionally, while reviewing material an undescribed *Vaabonbonphyllium* gen. nov. specimen was located and is herein described as *Vaabonbonphylliumrafidahae***gen. et sp. nov.** from Mt. Hagen, Papua New Guinea. Additionally, a morphologically unique clade of several species recovered as sister to the *Nanophyllium* sensu stricto was recognized and their numerous unique morphological features and monophyly leads the authors to erect the new genus *Acentetaphyllium***gen. nov.** which warrants the following new combinations: *Acentetaphylliumbrevipenne* (Größer, 1992), **comb. nov.**, *Acentetaphylliumlarssoni* (Cumming, 2017), **comb. nov.**, *Acentetaphylliummiyashitai* (Cumming et al. 2020), **comb. nov.**, and *Acentetaphylliumstellae* (Cumming, 2016), **comb. nov.** With the addition of several new genera, a key to phylliid genera is included for adult males and females.

## ﻿Introduction

**Figure 1.**
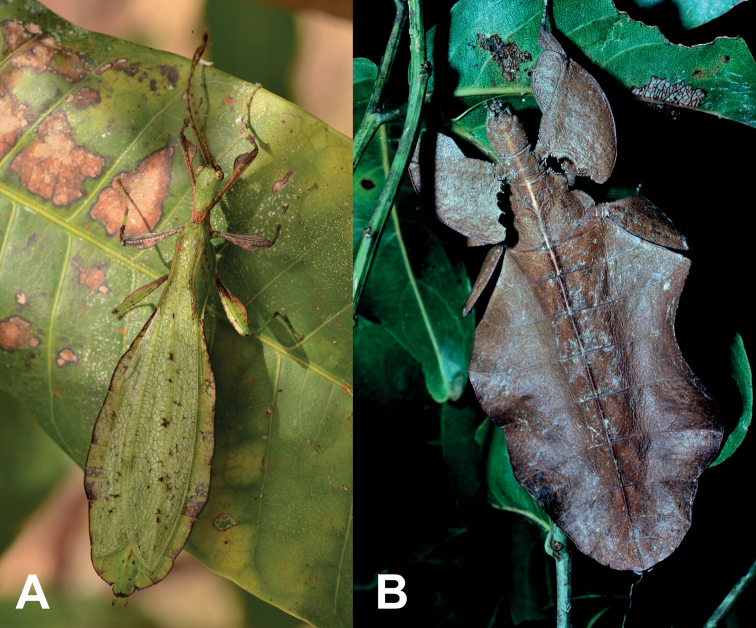
Live representatives from two of the three herein described genera **A***Rakaphylliumschultzei* comb. nov. male photographed by Loïc Degen (Switzerland) on Wokam Island, Aru Islands, Indonesia **B***Acentetaphylliumbrevipenne* comb. nov. brown form female, photograph purchased from Alamy stock photo website, noted as simply from the “rainforest of New Guinea”, no additional observational data could be traced.

The leaf insects (aka walking leaves) are masters of leaf masquerade, as both sexes have abdomen that are broad and thin with variable coloration to match their arboreal habitats (Fig. 1). Leaf insects are strongly dimorphic with males that are smaller and can fly (Fig. 1A) and females that are largely stationary, spending their lives in the canopy (Fig. 1B; Wedmann et al. 2007; Boisseau et al. 2021). This strong sexual dimorphism, coupled with their impressive camouflage means that leaf insects are unfortunately rather rare in collections despite having a significant range throughout most of Southeast Asia, and unless confirmed through molecular analyses or from captive rearing, pairing up the sexes is not always possible and can lead to significant identifications errors (Cumming et al. 2020a; Brock et al. 2021).

Due to their notable rarity within collections, some clades are poorly known (composed only of singular holotype specimens or few representatives within a couple museums around the world) and have therefore been somewhat neglected/lumped in with other, better-known clades based upon limited morphological features. Recently the first phylliid-wide molecular based phylogeny was completed which added a great deal of clarity to the generic relationships by digging beyond limited, few feature morphological evidence (Bank et al. 2021). Interestingly, a relationship which was uncovered within Bank et al. (2021) was that when observing a singular morphological feature between genera, certain relationships could be recovered that immediately became unsupported when a different singular morphological feature was reviewed. Additionally, in many cases these single morphological feature-based relationships did not agree with well-supported molecular based phylogenies (for example, in females the ventral color of the coxae can either be the same color as the surrounding tissue or a vibrant different variety of colors (like is seen in *Chitoniscus* Stål, 1875 and *Phyllium*Illiger 1798); however, these clades have not been recovered as sister to each other (Bank et al. 2021)). Instead, it has become apparent that phylliid relationships must be examined from multiple lines of evidence whenever possible (such as the erection of the *Trolicaphyllium*Cumming et al. 2021a from the *Chitoniscus* Stål, 1875 which was supported by numerous molecular based studies and 23 morphological characters; see Cumming et al. 2021a and citations therein).

Unfortunately, certain phylliid clades are so rare that they have yet to be sequenced to be included within molecular phylogenies leaving only morphology to reveal higher level relationships at this time. One area which appears to be highly diverse and significant for phylliid evolution is *Melanesia* (an area roughly encompassing New Guinea, Solomon Islands, Fiji, New Caledonia, and other nearby islands) as several genera have been described from this area in recent years and *Melanesia* was recovered as the likely ancestral range for all extant phylliids (Bank et al. 2021). With that in mind, several of the rarer and poorly known clades of *Melanesia* were systematically reviewed to correct their taxonomy to better reflect their recovered phylogenetic history.

All three of the herein described genera have previously been recognized (fully or partially) at one time or another as distinct to some degree. Within the substantial phylliid revision by Hennemann et al. (2009) their intrageneric systematizations recognized several Melanesian clades, namely the *schultzei* species group, *frondosum* species group, and the *brevipenne* species group (all of which at the time were nested within the *Pulchriphyllium* Griffini, 1898 sensu lato which was thought to be a subgenus of *Phyllium* Illiger, 1798). Most of the *frondosum* species group has since been found to be the previously unrecognized females to the *Nanophyllium* (suspected by Brock and Hasenpusch (2003); confirmed by Cumming et al. (2020a)) but the other clades have not yet been extensively reviewed and are therefore the focus of this work.

One group which is rare in collections is the *schultzei* group of *Pulchriphyllium* sensu lato, which were extensively reviewed because of their disjunct geographic distribution from the known *Pulchriphyllium* sensu lato range and the unique morphology of having a two lobed exterior protibia. Unfortunately, this clade has not been sequenced for molecular analyses yet, so only morphological review was possible. Interestingly numerous morphological features of this clade differentiate the *schultzei* group species from the *Pulchriphyllium* sensu stricto (such as the genitalia in females and alae wing venation in males). Additionally, within the species of the *schultzei* group, it was found that these same features which are disjunct from the *Pulchriphyllium* sensu stricto also separate the *schultzei* group into two morphologically distinct clades.

An additional species which has been included within molecular analyses was *Nanophylliumbrevipenne* (Größer, 1992), which was recovered as sister to all other sampled *Nanophyllium* Redtenbacher, 1906 species. Upon further review numerous morphological features were found that allow simple morphological distinction from the *Nanophyllium* and agree with the recovery of *brevipenne* and several related species within their own clade, sister to the *Nanophyllium*. This led the authors to review the genus concept with the phylliids to determine (based upon how other genera are classified within the phylliids) just how unique must a clade be to differentiate it taxonomically from another.

## ﻿Materials and methods

The following collection acronyms are used:

**BPBM**Bishop Museum,Honolulu, Hawaii, USA;

**Coll DG** Private collection of Detlef Größer, Berlin, Germany;

**Coll RC** Private collection of Royce T. Cumming, California, USA;

**Coll SLT** Private collection of Stéphane Le Tirant, Québec, Canada;

**Coll TM** Private collection of Tetsuo Miyashita, Japan;

**ANIC (CSIRO)** Australian National Insect Collection, Canberra, Australia;

**IMQC** Insectarium de Montréal, Montréal, Québec, Canada;

**MAMU**Macleay Collections,Sydney University Museums, Sydney, Australia;

**MNHN**Muséum National d’Histoire Naturelle,Paris, France;

**MNHU** Museum für Naturkunde der Humboldt-Universität, Berlin, Germany;

**NHMUK**Natural History Museum United Kingdom,London, United Kingdom;

**RBINS**Royal Belgian Institute of Natural Sciences,Brussels, Belgium;

**SDEI**Senckenberg Deutsches Entomologisches Institut, Müncheberg, Germany;

**SDNHM**San Diego Natural History Museum,California, USA;

**SMTD** Staatliches Museum für Tierkunde, Dresden, Germany;

**ZFMK** Zoological Research Museum Alexander Koenig, Bonn, Germany.

### ﻿Specimens

Specimens reviewed within this study are from various institutional and private collections and when specimens could not be reviewed in person, high-resolution images from The World of Stick Insects website (Phasmatodea.com) and the Phasmida Species Files Online (Brock et al. 2021) were reviewed. Specimen data and depositions are presented in Suppl. material 1 which was utilized to generate the distribution map (Fig. 5) and the discussion sections of each species herein reviewed.

### ﻿Phylogenetic analyses

As several important taxa that are the focus of this work are poorly known and presently lack molecular data for phylogenetic analyses, combined molecular and morphological matrices were generated and analyzed to provide as much support as possible but also include these rare taxa. Additionally, because several of the species of significance for this work are poorly known, the opposite sex associations are only based upon morphological similarities (but not yet confirmed through rearing (as was done in Cumming et al. (2020a) for *Nanophylliumasekiense* (Größer, 2002) or through molecular analyses like in Bank et al. (2021) for *Pseudomicrophylliumpusillulum* (Rehn and Rehn, 1934)). Therefore, phylogenetic analyses were carried out on separate male and female morphological matrices to take into consideration the possibility of erroneously associating the opposite sexes of different species which in these strongly sexually dimorphic insects is a possibility.

Where positively associated opposite sexes have been confirmed from past studies, molecular data from a single specimen (and therefore a single sex) was used for both male and female molecular datasets. For example, as *Pulchriphylliumpulchrifolium* (Audinet-Serville, 1838) is well-known and the opposite sex has been confirmed through captive rearing, the molecular data from only one specimen (in the case of this study, female specimen ID Coll RC 16-030) was utilized for both the male and female datasets molecular portion. All molecular data utilized for this study is that which was utilized within Bank et al. (2021) with GenBank accession numbers and specimen data outlined in Suppl. material 2. The molecular data was organized into 12 subsets based on three ribosomal genes (16 S, 18 S, 28 S) and three protein-coding genes (COI, COII, H3) resulting in a supermatrix of 4694 bp (see Suppl. material 3).

Morphological features with their codable states and the resulting morphological matrices are separated by sex and are outlined in Suppl. materials 4 and 5, respectively. Wherever possible the specimen which was originally sequenced within Bank et al. (2021) was reviewed for the morphological dataset, but when not possible/applicable specimens from the authors collections, museums, and online images were utilized.

As Phylliidae has repeatedly been recovered as monophyletic (Buckley et al. 2009; Robertson et al. 2018; Bank et al. 2021; Bank and Bradler 2022) and the focus of this work was on little known clades represented almost exclusively by morphological data, only one outgroup taxon was included to root our Phylliidae focused tree (see Suppl. material 2 for taxa details). As the focus of this work was on the arbitrary rank of genus, the focus of the morphological matrices was features which are commonly associated with generic differentiation and have been the focus of past revisionary works/keys to phylliid genera. The list of morphological features utilized is limited as the focus was not on the species level for morphological differentiation (which would require a great deal more fine detail characters) and only features considered significant for higher taxonomic level differentiation were included. In total, for both male and female analyses, there were 27 phylliid taxa for females and 28 males were included representing all currently known and herein described genera.

**Figure 2.**
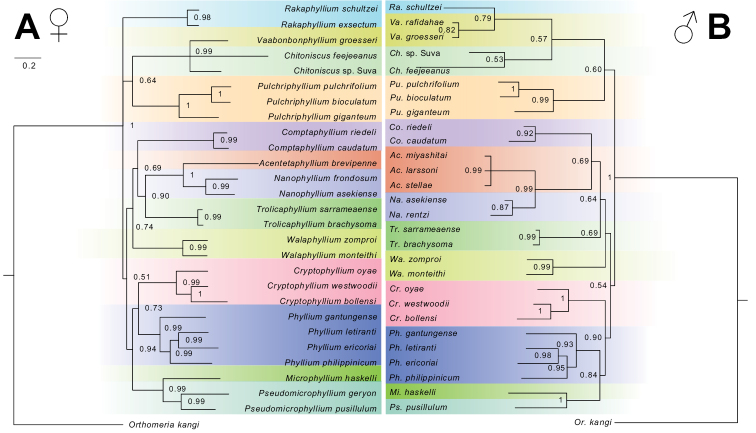
Phylogenetic reconstruction utilizing Bayesian Inference (BI) of representative taxa from all Phylliidae genera (morphology + DNA). Values presented are the posterior probability. Reconstruction based on a supermatrix of 4694 bp of molecular data and morphological characters (33 characters for females **A** and 25 characters for males **B** Branch length is proportional to relative divergence with the scale bar in the upper left indicating 0.2 units of divergence. Genera are color-coded to match between the female and male trees.

Phylogenetic trees (Fig. 2) were inferred via Bayesian inference (BI) performed using MrBayes v. 3.2.6 × 64 (Ronquist et al. 2012) implemented in the online CIPRES Science Gateway v. 3.3 (https://www.phylo.org, Miller et al. 2010). Two runs of four chains each were sampled for 30 million generations with samples taken every 5,000 generations with the first 25% of the samples discarded as burn-in. For additional parameters and summary statistics see Suppl. material 6. Resulting tree files were visualized and edited using Figtree v. 1.4.4 (https://github.com/rambaut/figtree/) and Adobe Illustrator v. 16.0.0 (Adobe Inc., San Jose, USA). Parsimony analyses were also conducted using TNT (Tree Analysis Using New Technology) v. 1.5 (Goloboff and Catalano 2016) made available by the Willi Hennig Society (Goloboff et al. 2008) downloaded for use on Windows. The search strategy to find the shortest trees used a combination of sectorial searches, ratchet (with the perturbation phase set to eight up-weighting probability and four down-weighting probability), drift, and tree fusing. The minimum length was set to be found 15 times and Tree Bisection and Reconnection (TBR) method of branch swapping was enacted to search the available tree space. Support values were estimated using 1,000 replicates for both the standard bootstrap and jackknife and these parsimony results are available in Suppl. material 7.

### ﻿Photography

**Figure 3.**
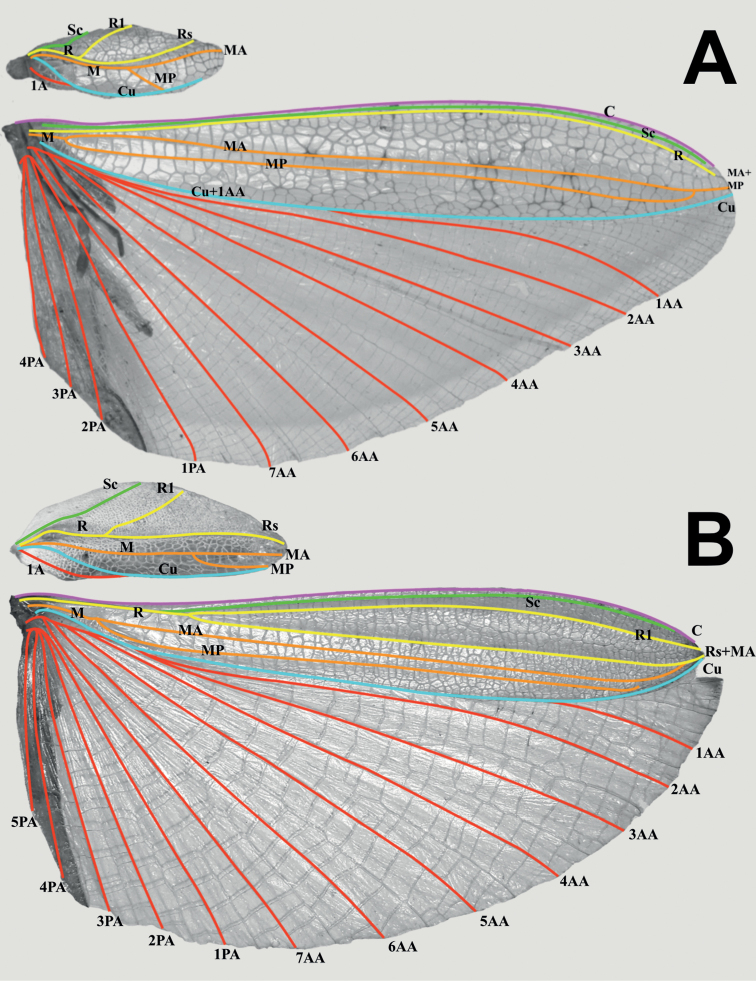
Male tegmina venation for two of the herein described genera **A***Rakaphylliumschultzei* comb. nov. (MNHU) **B***Vaabonbonphylliumrafidahae* (IMQC) (Coll RC 19-106). Abbreviations: Sc (subcosta); R (radius); R1 (radius 1); Rs (radial sector); Rs+MA (fused radial sector and media anterior); M (media); MA (media anterior); MP (media posterior); Cu+1AA (fused cubitus and first anterior anal); 1AA–7AA (first through seventh anterior anal); 1PA–5PA (first through fifth posterior anal); 1A (first anal).

**Figure 4.**
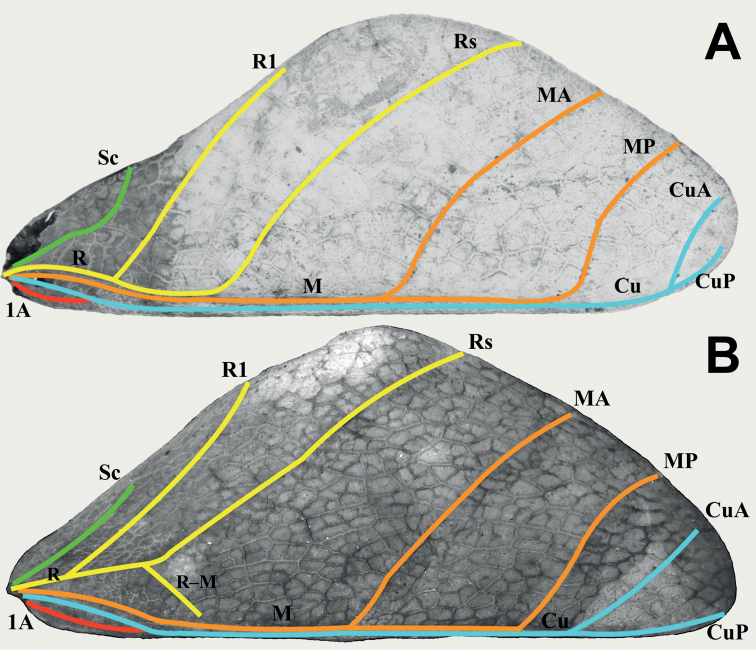
Female tegmina venation for two of the herein described genera **A***Rakaphylliumschultzei* comb. nov. (SDEI) **B***Vaabonbonphylliumgroesseri* comb. nov. (SMTD). Abbreviations: Sc (subcosta); R (radius); R1 (radius 1); Rs (radial sector); R–M (radius to media crossvein); M (media); MA (media anterior); MP (media posterior); Cu (cubitus); CuA (cubitus anterior); CuP (cubitus posterior); 1A (first anal).

Photographs of specimens deposited within the IMQC, Coll SLT, and Coll TM were taken by René Limoges (IMQC) using a Nikon D850 DSLR camera (Nikon Corporation, Tokyo, Japan) with Nikon Micro-Nikkor 200 mm f/4 lens on Manfrotto 454 micrometric positioning sliding plate (Manfrotto, Casolla, Italy). Lighting was provided by two Nikon SB-25 flash units with a Cameron Digital diffusion photo box (Henry’s, Vancouver, Canada). Photographs of specimens within the first authors collection (Coll RC) were taken by the first author using a Canon 5D Mark II and a MP-E 65 mm macro lens and stacked using Zerene Stacker (Zerene Systems LLC, Richland, USA). Photographs not taken by René Limoges (IMQC) are accompanied by citation to their photographers and were taken using a variety of equipment. All images were edited using Adobe Photoshop Elements 13 (Adobe Inc., San Jose, USA) to remove their backgrounds and correct brightness/contrast. For wing illustrations (Figs 3, 4) specimens were chosen with the tegmina and alae already pinned flat, and the wings were photographed and simply cropped in the photos from the bodies of the specimens, not dissected from the specimens. Wing venation terminology follows Burt (1932) and Ragge (1955).

### ﻿Morphological abbreviations (listed morphologically anterior to posterior)

**C** costa

**Sc**subcosta

**R** radius

**R1** radius 1

**Rs** radial sector

**R–M** radius to media crossvein

**M** media

**MA** media anterior

**MP** media posterior

**MA+MP** fused media anterior and media posterior

**Rs+MA** fused radial sector and media anterior

**Cu** cubitus

**CuA** cubitus anterior

**CuP** cubitus posterior

**Cu+1AA** cubitus and first anterior anal

**1A** first anal

**1AA–7AA** first–seventh anterior anal

**1PA–5PA** first–fifth posterior anal

## ﻿Results

Both the parsimony analyses based solely upon morphology data (Suppl. material 7) and the Bayesian analyses based upon the combined molecular and morphological datasets (Fig. 2) gave similar relationships regarding the taxa of focus. However, when reviewing these results, it should be remembered that two of the clades of focus are only represented by morphological data (therefore in the combined molecular and morphology datasets a great deal of information is lacking). Despite this, the within clade relationships were almost always strongly supported and only the deeper recovered relationships were less reliable (Fig. 2).

**Figure 5.**
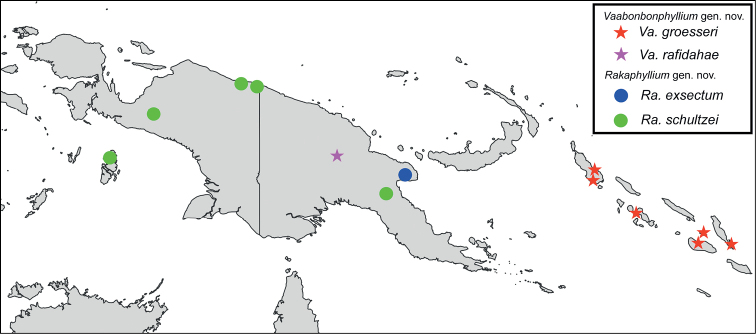
Distribution map noting all presently known records of *Vaabonbonphyllium* gen. nov. (denoted as star symbols) and *Rakaphyllium* gen. nov. (denoted as circle symbols) which were located and had data that could accurately be mapped. See Supplementary File 1 for full details for all records presented.

Most of the species of focus for this study were only represented by morphological data which presents a challenge. This is because most morphological features when viewed singularly appear to unite a subset of clades, but as soon as a different feature is reviewed a different subset of clades appear more closely linked (see the supplementary discussion in Bank et al. 2021 for further discussion on this point). This creates clades which, when lacking molecular support, have notably low support values. Interestingly though even within the analyses of Bank et al. (2021) which only included species which had molecular data, some clades still showed somewhat low support values.

**Figure 6.**
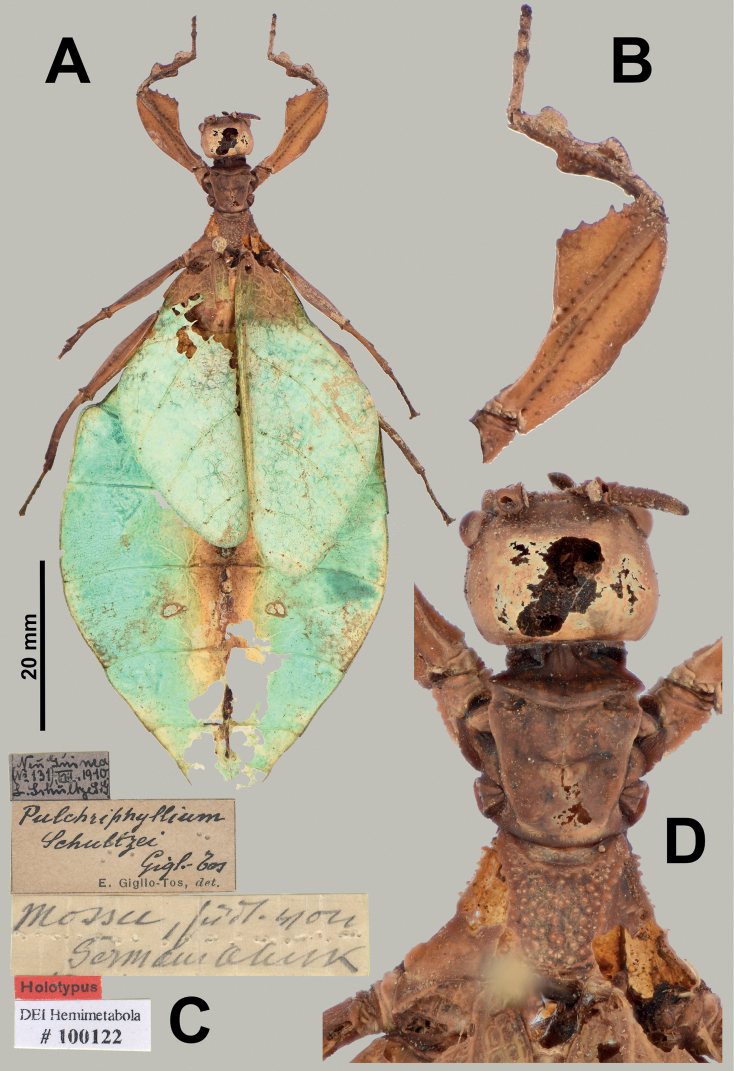
*Rakaphylliumschultzei* comb. nov. holotype female. Note that the holotype female is heavily damaged in several places, especially the abdomen which has numerous holes through which the background can be seen, this is not an artifact of image postprocessing **A** habitus, dorsal **B** right front leg, dorsal **C** associated data labels **D** details of head through thorax, dorsal. Scale bar only associated with **A**. Photographs by Arne Köhler (SDEI) and Mandy Schröter (SDEI).

**Figure 7.**
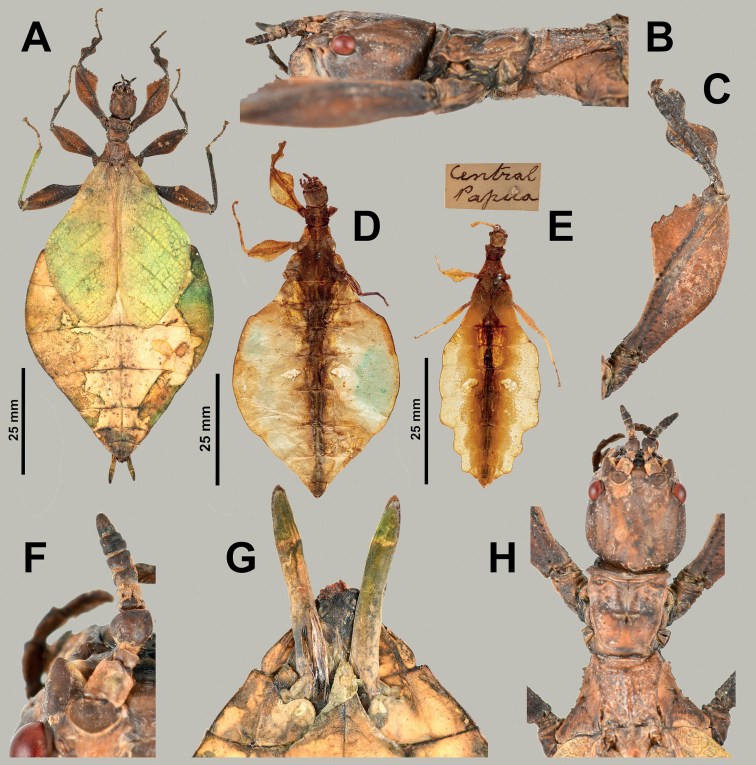
*Rakaphylliumschultzei* comb. nov. non-type material specimens **A–C, F–H** adult female from Coll SLT, Indonesia: Jayapura, Klaisu **D, E** nymphs from MAMU**A** habitus, dorsal **B** details of head through thorax, lateral **C** profemora, dorsal **D** female nymph without collection data (MAMU) **E** male nymph with slightly undulating abdomen with associated data label above (MAMU) **F** details of antenna, dorsal **G** genitalia, ventral **H** details of head through thorax, dorsal. Scale bars only associated with images **A, D, E**. Photographs **A–C, F–H** taken by Rene Limoges (IMQC) **D, E** courtesy of Chau Chak Wing Museum, the University of Sydney, and photographs taken by David James.

Within the male-based analyses of this study, *Pulchriphylliumschultzei* Giglio-Tos, 1912 was recovered as sister to a clade formed by *Pulchriphylliumgroesseri* (Zompro, 1998) and an undescribed species with moderately high support (Fig. 2). Interestingly, within the female-based analyses *Pulchriphylliumgroesseri* was instead recovered as sister to the *Chitoniscus* Stål, 1875 while *Pulchriphylliumschultzei* and *Pulchriphylliumexsectum* (Zompro, 2001b) were recovered as external to this clade and instead were part of a polytomy deeper within the recovered phylogeny (Fig. 2). The important takeaway from these analyses is that historically the species *Pulchriphylliumgroesseri*, *Pulchriphylliumschultzei*, and *Pulchriphylliumexsectum* are considered to belong within the *Pulchriphyllium* sensu lato simply due to the presence of exterior tibial lobes (regardless of the fact that *Pulchriphyllium* sensu stricto has a single large lobe and these other species have two smaller lobes; Fig. 7C). The results of these phylogenetic analyses instead place these “two-lobed” species as distinctly outside of the reviewed *Pulchriphyllium* sensu stricto species. Therefore, as the taxonomy currently stands, *Pulchriphyllium* sensu lato is polyphyletic when the morphologically unique species *Pulchriphylliumgroesseri*, *Pulchriphylliumschultzei*, and *Pulchriphylliumexsectum* are included.

Additionally, in reviewing the *Nanophyllium* sensu lato, this distinct clade was recovered as bifurcate with significant divergence distance between two internal sister clades. These sister clades correspond to two morphologically distinct groups which have been recognized by past authors as significant (Hennemann et al. 2009; Cumming et al. 2020a). Essentially taxonomic ranks above the species level are arbitrarily assigned in order to facilitate communication about natural groups, therefore these two morphologically distinct sister clades based upon the current treatment of genera within the phylliids clearly align themselves with the genus concept well. A designation which can facilitate future species differentiation within either of these clades.

In summary, the phylogenetic analyses recovered two distinct clades which are currently taxonomically treated as *Pulchriphyllium* sensu lato members and one morphologically unique clade which is sister to the *Nanophyllium* sensu stricto. All three of these clades are morphologically unique to an extensive degree (with such significant differences that within the phylliids such levels of morphological uniqueness are treated by taxonomists as genera) and therefore in order to recognize these three clades with autapomorphic morphological features as unique and to correct the current polyphyly, the following three genera are erected below.

### ﻿Taxonomy

**Phylliinae Brunner von Wattenwyl, 1893**

#### Phylliini Brunner von Wattenwyl, 1893

##### ﻿ Rakaphyllium gen. nov.

Taxon classificationAnimaliaPhasmatodeaPhylliidae

8AF2A235-E439-5123-82B0-03F2FF646446

https://zoobank.org/2C2C6B6B-83F4-4341-954C-351E92119BEA

###### Type species.

*Pulchriphylliumschultzei* Giglio-Tos, 1912: 56, herein designated.

###### Taxonomic hierarchy.

This genus has a combination of features which link it to several genera, thus making a higher-level taxonomic placement difficult and requiring molecular confirmation in the future. The thorax and tegmina venation in the females are reminiscent of some *Pulchriphyllium* sensu stricto species and the profemoral and protibial lobes are reminiscent of some *Phyllium* Illiger, 1798 and *Comptaphyllium*Cumming et al., 2019 species, whereas the male thorax is similar to *Trolicaphyllium* Cumming et al., 2021. At present within Phylliidae there are two recognized tribes, the NanophylliiniZompro and Größer 2003 (which contains only the *Nanophyllium* Redtenbacher, 1906) and the Phylliini Brunner von Wattenwyl, 1893 (which contains all other genera). Therefore, at this time this genus is placed within the tribe Phylliini as notable features such as a two lobed posteromedial tubercle and a prescutum which is wider than long (features which help to define the *Nanophyllium*) are absent, suggesting a closer relationship to other genera instead.

###### Discussion.

The selected type species for this new genus is *Pulchriphylliumschultzei* Giglio-Tos, 1912 (= *Rakaphylliumschultzei* (Giglio-Tos, 1912), comb. nov.) as this was the first species described within this new genus, the holotype is from an exact collection locality, and this species appears to be the more commonly encountered of the two species within this new genus.

This new genus has been recognized as unique in the past, as this clade was designated as the *schultzei* species group within Hennemann et al. (2009) based upon the shorter tegmina and the two lobes on the exterior protibiae. While these features are helpful to differentiate this genus from others, the below noted autapomorphic features allow differentiation from all phylliid genera.

###### Autapomorphic features.

Within each sex there is an easily observed morphological feature which supports their monophyly and readily separates them from other phylliid genera. For females, the gonapophyses VIII are exceptionally long, with approximately half of their length projecting from under the terminal abdominal segment (Figs 7G, 9D), a feature not seen in any other phylliid as typically gonapophyses VIII only reach to the apex of the terminal abdominal segment or at most only exceed the tip slightly (Fig. 10E). For males the alae venation is unique as the radius vein is simple (Fig. 3A), not bifurcate as is seen in all other phylliid genera (Fig. 3B). These autapomorphic features help to define the new genus *Rakaphyllium* gen. nov. within the phylliids as well as differentiate them from the *Pulchriphyllium* sensu stricto.

###### Generic characteristics.

The *Rakaphyllium* gen. nov. are average sized phylliids, with females ranging from ca. 80 mm to 90 mm long and males ca. 60 mm long. Typical coloration appears to be green, but with so few specimens known and color variation a common occurrence in phylliids it is possible that this genus may also exhibit color forms (such as possibly *Rakaphylliumexsectum* comb. nov. but this brown coloration may simply be due to the age and preservation technique (Fig. 9)).

***Antennae*.** Females have antennae with nine segments with the terminal antennomere not notably long (only as long as the previous one to two segments combined) and segments IV through VIII all of a similar length (Figs 6D, 7F, 9E). Males have antennae which range from 20 to 23 segments with most segments covered densely in setae, and overall antennomere shape somewhat flattened.

**Figure 8.**
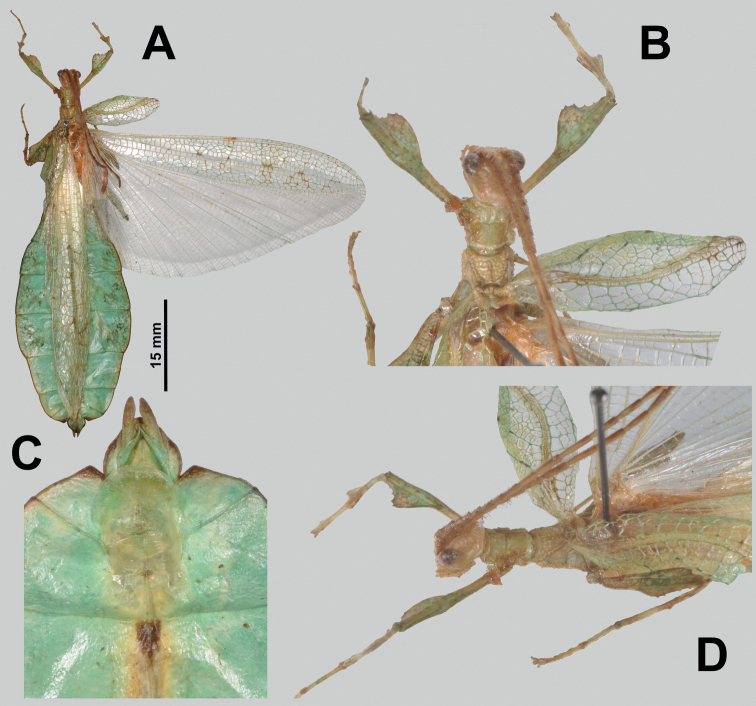
*Rakaphylliumschultzei* comb. nov. male from the MNHU collection **A** habitus, dorsal **B** details of front legs, base of antennae, head, thorax, and tegmina, dorsal **C** genitalia, ventral **D** details of front legs, base of antennae, head, thorax, and tegmina, dorsolateral. Scale bar only associated with **A**. Photographs by Frank Hennemann (Germany).

***Head capsule*.** Males have well-developed ocelli (Fig. 8B), females do not have ocelli (Figs 6D, 7H). Males have compound eyes which are strongly protruding and occupy ca. 2/5 of the head capsule lateral margins (Fig. 8B) versus females which have compound eyes which are notably smaller, only occupying less than 1/3 of the head capsule lateral margins and which do not strongly protrude from the capsule (Fig. 7H). Both sexes have head capsules which are marked by irregularly sized and space granulation.

**Figure 9.**
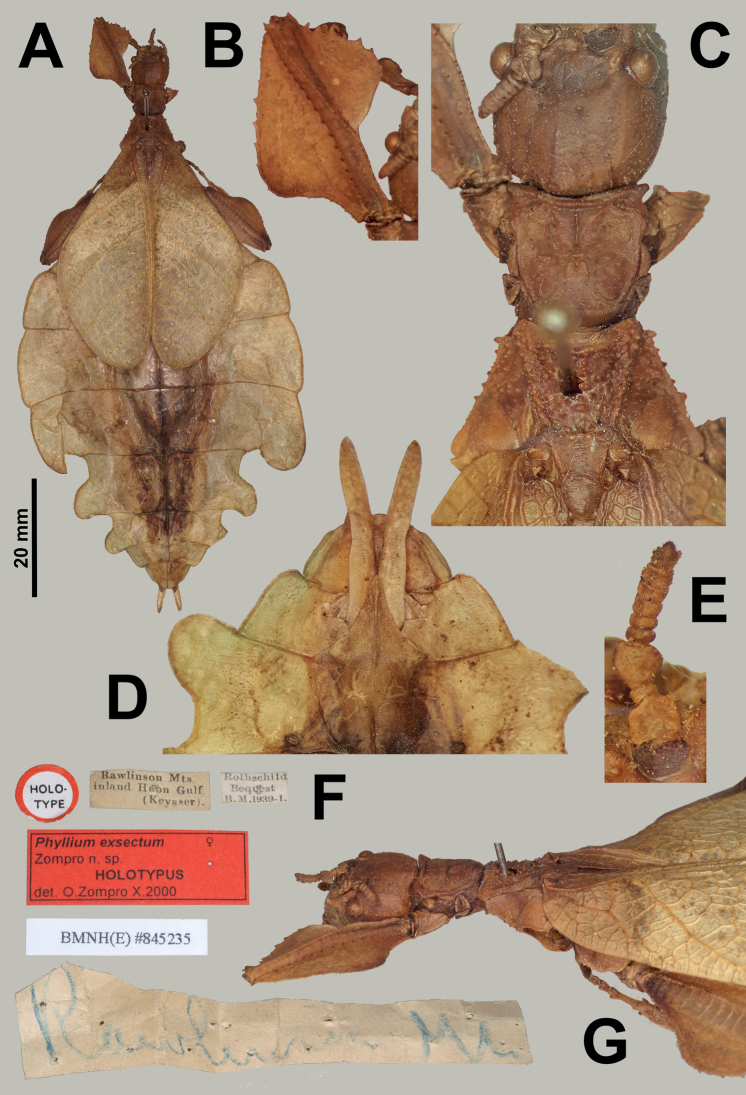
*Rakaphylliumexsectum* comb. nov. holotype female (NHMUK) **A** habitus, dorsal **B** profemora, dorsal **C** details of head through thorax, dorsal **D** genitalia, ventral **E** details of antenna, dorsal **F** associated data labels **G** details of head through thorax, dorsolateral. Scale bar only associated with **A**. Photographs **A, F, G** taken by Paul Brock (United Kingdom), other photographs taken by first author.

***Thorax*.** The thorax is similar in both sexes with mesopleurae that are narrowly diverging from the anterior to the posterior (evenly so in females, almost parallel in males for the anterior half and then more prominently on the posterior half) are marked on the anterior half with three to four small tubercles with granulation interspersed with the posterior half relatively smooth or with only minimal granulation (Figs 6D, 7H). In both sexes the prescutum is about the same length as the width of the anterior margin, with a posterior margin that is slightly narrower giving the prescutum a slight isosceles trapezoid appearance. The margins of the prescutum are marked with granulation and the prescutum surface is covered with granulation with those along the sagittal plane slightly larger and in males a weak sagittal crest is present (Figs 6D, 8B, 9C). When viewed laterally, both sexes have a weakly formed prescutum anterior rim with a granular surface (Figs 7B, 8B).

***Legs*.** Both sexes have interior protibial lobes which do not span the full length of the shaft, instead they are only situated on the proximal half (Figs 6B, 7C) and exterior protibiae which are marked by two lobes, one on the proximal and one on the distal end (Figs 6B, 7C). The exterior meso- and metatibiae are notably reduced, but if lobes are present its just as small spurs (sometimes just a distal spur or sometimes one on each end of the shaft) never as prominent lobes. Profemoral exterior lobes are rather variable as within *Rakaphylliumschultzei* comb. nov. females they are simply arcing smoothly from end to end without a strong angle (Fig. 6B), but in *Rakaphylliumexsectum* comb. nov. females the exterior lobe is distinctly boxy with a right angle (Fig. 9B). Males are only known for *Rakaphylliumschultzei* comb. nov. in which the femoral morphology is similar to the female, simply arcing tightly along the profemoral shaft (Fig. 8B).

***Wings*.** Female tegmina are average in length, only reaching onto abdominal segments V or VI and male tegmina are moderate in length, only reaching onto abdominal segment II or III. Females have rudimentary alae. Male alae are fully developed in an oval-fan configuration and reach onto abdominal segments VIII to X. Female tegmina have a subcoastal vein; radial vein which runs parallel with the media and splits into the first radial about halfway through its length and terminates in a radial sector which bends distinctly away from the media and arcs to the wing margin; a bifurcate medial vein; a bifurcate cubitus vein; and a first anal vein which fuses with the cubitus early on (Fig. 4A). Male tegmina (Fig. 3A) have a simple subcoastal vein; radial vein which runs parallel/subparallel with the media almost throughout the full length of the wing and branches into the first radial about one third of the way through the wing length and terminates as the radial sector; the media runs parallel/subparallel with the radius and has the media posterior split near the middle of the wing and terminates as the media anterior; the cubitus is unbranched; and there is a first anal which fuses with the cubitus early on. Male alae (Fig. 3A) have a costal vein running along the anterior margin; a subcostal vein which runs parallel with the costal vein for the full length; the radial vein is the most unique feature of the alae as it is simple, running parallel to the subcostal vein; the media splits early into the media anterior and posterior which run parallel until the media posterior fuses with the media anterior near the wing margin and they run fused to the apex of the wing; the cubitus is fused with the first anterior anal for ca. half of the length until the first anterior anal splits and runs to the wing margin; the anal veins are split into two groups, the anterior anals and the posterior anals (with seven anterior anals and four or five posterior anals).

***Abdomen*.** Both sexes have variable abdominal shapes, but all forms are broad in the middle with the widest segments V or VI; in both sexes the anterior halves are uniformly broadening to the middle segments, and then the posterior half of the abdomen is variable either with smooth margins giving the abdomen an ovoid appearance, or with the posterior segments gently or strongly undulating giving the abdomen a lobed appearance. Female subgenital plate is short and relatively narrow with the apex slightly reaching onto the anterior margin of the terminal abdominal segment and ending in a fine point; gonapophyses VIII are exceptionally long (with ca. half of their length projecting out from underneath the abdomen) with a uniform width through most of their length; the cerci are relatively flat, marked sparsely with a granular surface, and end in blunt points (Figs 7G, 9D). Males have a long, relatively narrow, triangular vomer which is singularly pronged, hooking up into the paraproct (Fig. 8C).

***Egg.*** Egg morphology is not yet known for this rare genus, but with such unusually long gonapophyses to hold the eggs before they are flung away, the eggs must have a unique shape to require such ungainly gonapophyses.

###### Etymology.

*Rakaphyllium* meaning “walking leaf”. This generic epithet is a compound of the Latinized name *Phyllium* the type genus for the family (from Greek φυλλον, -ου (*phyllon*, -*oy*) + -um; Poitout 2007), coupled with the prefix *raka* from the Hiri Motu language of New Guinea which means “to walk” (Chatterton 1975; Dutton and Voorhoeve 1975). We wish to honor the original inhabitants of this area by using the traditional language of Hiri Motu which is one of the official languages of Papua New Guinea and as Papua New Guinea pushed towards sovereignty in 1975, Hiri Motu was seen as a unifying force which was instrumental in the awakening of national pride (Dutton and Voorhoeve 1975). We chose this name because of the amazing camouflage these insects possess, allowing them to appear miraculously as a leaf that simply stands up and walks away when disturbed. This new genus is neuter in gender, following *Phyllium*.

###### Distribution.

At present our knowledge of the *Rakaphyllium* gen. nov. is rather limited due to its rarity, however, interestingly despite it being rarely collected, this genus appears to be somewhat widespread, with records from throughout New Guinea, and even a record from the Aru Islands (Fig. 1A) off the western coast of Papua Province, Indonesia (Fig. 5).

### ﻿New combinations

***Rakaphylliumschultzei* (Giglio-Tos, 1912), comb. nov.**

#### *Rakaphylliumexsectum* (Zompro, 2001b), comb. nov.

##### ﻿ Rakaphyllium schultzei

Taxon classificationAnimaliaPhasmatodeaPhylliidae

(Giglio-Tos, 1912) comb. nov.

2C6EA639-BB83-520A-B18C-4381C8D6877A

Figs 1A , 3A , 4A , 6 , 7 , 8

###### Material examined.

(3 ♀♀, 3 ♂♂, 3 ♂♂ nymphs, 2 ♀♀ nymphs): ***Holotype*** (♀): “New Guinea (No 131) VIII. 1910 L. Schultze L.J.; *Pulchriphylliumschultzei* Giglio-Tos, E. Giglio-Tos, det.; Mossu südl von Germainhuk; Holotypus; DEI Hemimetabola #100122” (SDEI; Fig. 6). See Suppl. material 1 for additional specimens reviewed, their collection data, and depositories.

###### Remarks.

This rarely encountered species was described from a female from northern New Guinea (Fig. 6), and since its description over a century ago few additional specimens have been collected. At present due to the rarity of material and lack of fresh material for molecular comparison, the male specimens associated with this species are only assumed based on shared morphology (Fig. 8) but have not been confirmed yet.

###### Differentiation.

For female *Rakaphylliumschultzei* comb. nov., the abdominal shape and profemoral lobes easily differentiate it from *Rakaphylliumexsectum* comb. nov. Within *Rakaphylliumschultzei* comb. nov. the profemoral exterior lobe arcs smoothly from end to end without a strong angle (Fig. 6B), but in *Rakaphylliumexsectum* comb. nov. the exterior lobe is distinctly boxy with a right angle (Fig. 9B). Additionally, abdominal shape appears to be reliable for differentiation, but some abdominal variation has been observed in a few *Rakaphylliumschultzei* comb. nov. females to include perfectly smooth margins and some with slight undulations on the terminal abdominal segments (although none have been observed to be as extreme as in *Rakaphylliumexsectum* comb. nov.) therefore abdominal shape may also be useful for differentiation.

###### Distribution.

At present we are aware of records from several locations throughout New Guinea (both on the Indonesian and Papuan sides) and records from the Aru Islands which visually appear to be this species despite the geographic disconnect from mainland New Guinea (see Fig. 5 and Suppl. material 1 for details of these records). The distribution of this species is vague as a thorough knowledge of morphological variation (due to limited material) and molecular analyses are lacking at present, and therefore our identification assumptions are only based upon general morphology. Unfortunately, without positive confirmation of the males we assume are this species, this distribution may be over expansive (possibly representing several species instead of one), and especially for the observational record from the Aru Islands (Fig. 1A), the specimen could not be examined and although the habitus appears to match the morphology of the mainland New Guinea male the fine details may suggest otherwise if a specimen could be examined.

##### ﻿ Rakaphyllium exsectum

Taxon classificationAnimaliaPhasmatodeaPhylliidae

(Zompro, 2001b) comb. nov.

AAE955A2-E02F-5470-8E1A-DCB6CCCE4765

Fig. 9

###### Material examined.

***Holotype*** (♀): “HOLO-TYPE; Rawlinson Mts. inland Huon Gulf. (Keysser).; Rothschild Bequest B. M. 1939-I.; Rawlinson Mt; *Phylliumexsectum* ♀ Zompro n. sp. HOLOTYPUS det. O. Zompro X.2000; BMNH(E) #845235” (NHMUK; Fig. 9).

###### Remarks.

This remarkable looking species is unfortunately only known from the singular holotype female from the little accessed Rawlinson Mountains of the Huon Peninsula (Fig. 5). Even in other phylliid species with prominent lobes on the abdomen, they pale in comparison to the unique habitus of this species, which likely evolved to mimic some strongly lobed host plant. Unfortunately, as this is the only specimen known and it lacks ecological data, nothing is known about a possible plant that the unique shape of this species may have evolved to resemble through advergent evolution.

###### Differentiation.

At present only the female is known for this species, no tentative males have been found which could represent the opposite sex. This species can be differentiated from its single other congenic by the elaborate lobed abdomen (although some *schultzei*-like females have been observed with slight lobes suggesting morphological plasticity within that species) or more reliably by the profemoral exterior lobe, which in *Rakaphylliumexsectum* comb. nov. is right angled (Fig. 9B), versus *Rakaphylliumschultzei* which always has the profemoral exterior lobe roundly arcing from end to end without a distinct angle present (Fig. 6B).

###### Distribution.

The type locality of Rawlinson Mts. on the Huon Gulf, Morobe Province, Papua New Guinea is the only record known at present (Fig. 5).

#### Phylliinae Brunner von Wattenwyl, 1893

**Phylliini Brunner von Wattenwyl, 1893**

##### ﻿ Vaabonbonphyllium gen. nov.

Taxon classificationAnimaliaPhasmatodeaPhylliidae

D5672784-A453-5663-9FCD-54275D0944B3

https://zoobank.org/0D8A7944-2461-4FFF-A041-A3A90E696398

###### Type species.

*Phylliumgroesseri* Zompro, 1998: 159, herein designated.

###### Taxonomic hierarchy.

Several features link this genus to the Phylliini genera and not the Nanophylliini, such as males with the alae radius split near the wing base (not splitting on the distal half of the wing as in *Nanophyllium* and *Acentetaphyllium* gen. nov.), females with tegmina venation similar to *Chitoniscus* due to the diverging radius and media veins and distinct cubitus anterior and cubitus posterior (cubitus only weakly bifurcate or not bifurcate in *Nanophyllium*), and both sexes have a singular posteromedial head tubercle (not bilobed as in *Nanophyllium* or *Acentetaphyllium* gen. nov.). Due to these notable morphological features, we place this herein described genus within the tribe Phylliini Brunner von Wattenwyl, 1893.

###### Discussion.

The selected type species for this new genus is *Phylliumgroesseri* Zompro, 1998 (= *Vaabonbonphylliumgroesseri* (Zompro, 1998), comb. nov.) as this was the first species described from within this new genus and this species is well represented by multiple specimens from the geographically isolated Solomon Islands (Fig. 4). Unfortunately, fresh specimens of either species within this genus have not yet been sequenced so hopefully this species can be collected one day and sequenced to add clarity to the higher-level taxonomy.

###### Autapomorphic features.

Within females, the first radial vein (R1) of the tegmina splits from the radius early on, ca. one third of the way between the wing base and the radius to media crossvein (R–M)/the bend in the radial sector (Fig. 4B) versus all other phylliids which have the first radius splitting from the radius midway or more than midway from the wing base to the radius to media cross vein (R–M)/the bend in the radial sector or even after this point.

For males, the media (M) vein of the tegmina runs parallel with the radial sector (Rs) several vein widths away and the media posterior (MP) splits on the distal half of the tegmina, bends immediately and runs parallel with the media anterior (MA) to the wing apex (Fig. 3B) versus in other genera typically the media and radial veins run side by side touching or nearly so, and the posterior branch(es) of the media split on the proximal half or near the middle of the length and run diverging (not parallel) from the media posterior and run to the wing margin, not the apex (Fig. 3A).

###### Generic characteristics.

The *Vaabonbonphyllium* gen. nov. are small to medium, with females ca. 70.0 mm in length (in *Vaabonbonphylliumgroesseri* comb. nov.) and with males from ca. 43.0 mm (*Vaabonbonphylliumgroesseri* comb. nov.) to ca. 53.0 mm (in *Vaabonbonphylliumrafidahae* gen. et sp. nov.).

**Figure 10.**
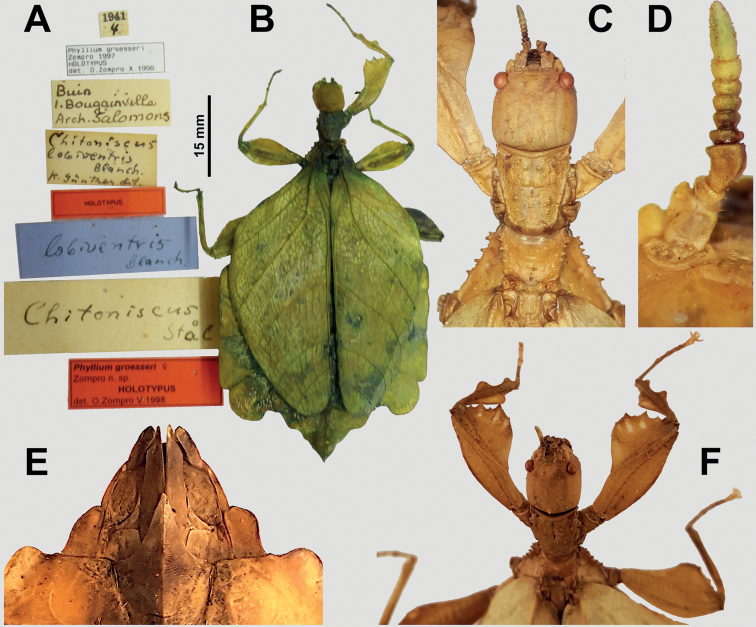
*Vaabonbonphylliumgroesseri* comb. nov. females **A** data labels associated with the holotype **B** holotype, habitus, dorsal **C** details of head through thorax, dorsal **D** details of antennae, dorsal **E** details of genitalia, ventral **F** front legs, head, and thorax, anterodorsal. Scale bar associated with **A** and **B**. **A** and **B** photographs by Christian Schmidt (SMTD), other images taken by the first author of specimens from the NHMUK.

**Figure 11.**
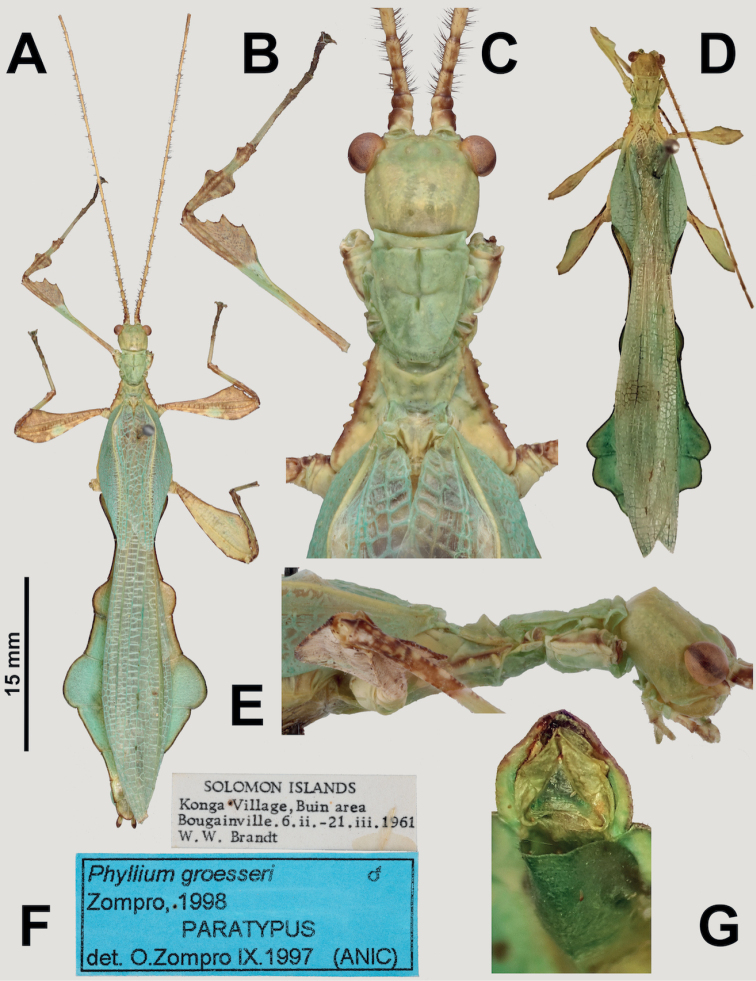
*Vaabonbonphylliumgroesseri* comb. nov. males **A–C, E, F** paratype male, (ANIC (CSIRO)), photographs by Bonnie Koopmans (CSIRO) **D, G** non-type male (NHMUK), photographs by first author **A** habitus, dorsal (left front leg is physically detached, photoshopped onto the specimen for this figure) **B** left front leg, dorsal **C** details of head through thorax, dorsal **D** habitus, dorsal **E** details of head through thorax, lateral **F** associated data labels **G** genitalia, ventral. Scale bar only associated with **A**.

***Antennae*.** Females have antennae with nine segments with segments I, III, and IV notably broader than the other segments and the terminal antennae segment is as long as the preceding three and a half segments (Fig. 10D). Males have antennae with around 22 segments (in *Vaabonbonphylliumgroesseri* comb. nov. both known male specimens have broken antennae that are missing segments, therefore they only have 19 or 20 segments, but in *Vaabonbonphylliumrafidahae* gen. et sp. nov. there are 22 segments that are not broken; Fig. 11F) and most segments covered in setae which are longer than the segment is wide.

**Figure 12.**
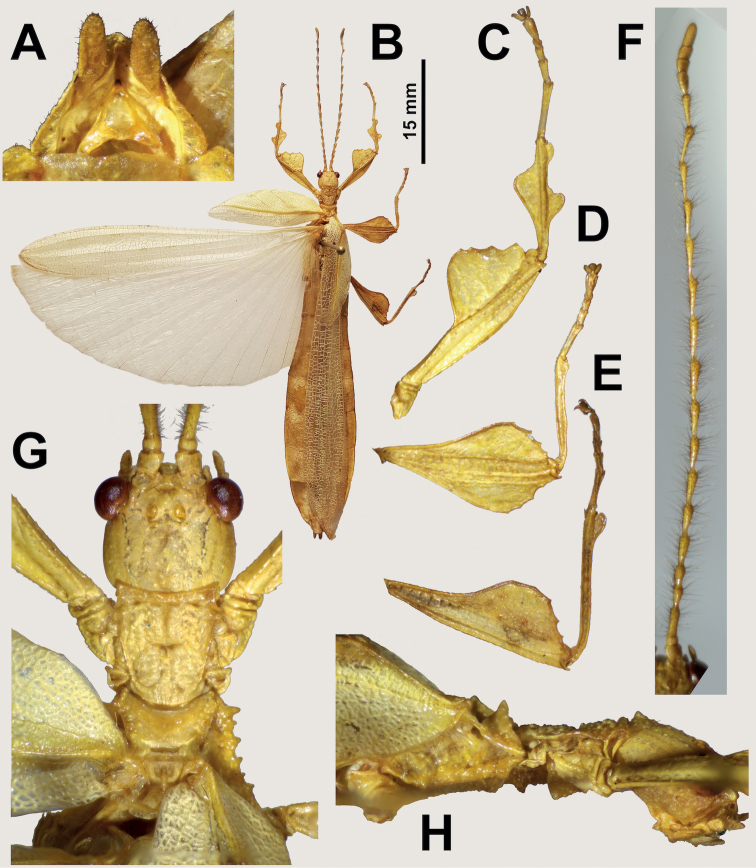
Holotype male *Vaabonbonphylliumrafidahae* gen. et sp. nov., (IMQC) (Coll RC 19-106) photographs by the first author **A** genitalia, ventral **B** habitus, dorsal **C** details of the protibial and profemoral lobes **D** details of the mesotibial and mesofemoral lobes **E** details of the metatibial and metafemoral lobes **F** antenna, dorsal **G** details of the head and thorax, dorsal **H** head–thorax, lateral. Scale bar only associated with **B**.

***Head capsule*.** Males have well-developed ocelli (Figs 11C, 12G) and female lack ocelli (Fig. 10C). Males have compound eyes which are strongly protruding and occupy ca. 2/5 of the head capsule lateral margins (Fig. 11C) versus females which have compound eyes which are notably smaller, only occupying ca. ¼ of the head capsule lateral margins and which to not strongly protrude from the capsule (Fig. 10C). In both sexes the head capsules are marked throughout by weak (Fig. 10C) to distinct granulation (Fig. 12G) which is relatively evenly spaced. The posteromedial head tubercle is singular and distinctly raised from the head capsule (Figs 11E, 12H).

***Thorax*.** The thorax is similar in both sexes with the pronotum notably longer than wide (Figs 10C, 11B, 12G). The mesopleurae are gradually diverging from the anterior to the posterior and are marked with four to six tubercles and some minor granulation throughout (Figs 10C, 11B, 12G). In both sexes the prescutum is ca. 2X wider on the anterior than long with lateral margins marked by three to five nodes/tubercles, and a prescutum surface which can be relatively smooth or slightly granular. When viewed laterally, both sexes have the prescutum anterior rim marked with a raised sagittal spine (Fig. 12H).

***Legs*.** Both sexes have interior tibial lobes on the protibiae which do not span the full length of the shaft (only occupying the proximal ½ to 4/5, but not fully reaching), have two lobes on the protibial exterior (one on the distal end which is smaller and one near the middle which is larger; Figs 10F, 11B, 12C), and the meso-, metatibiae are mostly bare except for the exterior distal ends which can be variable, by either being slightly raised or with small to medium sized lobes present (Fig. 12D, 12E). In both sexes the profemoral interior lobe is broader than the exterior lobe and marked with three or four broad teeth which can be more sharply pointed (Fig. 11B) or dulled (Fig. 12C). Both sexes have the interior mesofemoral lobes slightly broader or about even in width to the exterior lobes, and both lobes can have serration on the distal ½ to 1/3 of the lobe, but the interior lobe serration is more prominent than the exterior lobe. Both sexes have the interior metafemoral lobes several times broader than the exterior lobes and the interior lobes are prominently marked by serration.

***Wings*.** Female tegmina are always long, reaching onto abdominal segments VIII or IX and male tegmina are moderate in length, reaching onto abdominal segment III. Female alae are highly reduced to no more than just a nub and male alae are fully developed in an oval-fan configuration reaching slightly past the apex of the terminal abdominal segment. Female tegmina have a subcostal (Sc) vein which is simple and runs parallel with the wing margin for ca. ⅕ of the wing length before fading; radial vein which diverges steadily away from the media and splits into the first radial vein (R1) which diverges from the radius (R) early on, ca. ⅓ of the way between the wing base and the radius to media crossvein (R–M)/the bend in the radial sector, the primary vein terminates as the radial sector (Rs) on the wing margin just past the middle of the wing length, and as a small radial to medial crossvein (R–M) which does fully connect; the media vein (M) runs side by side the cubitus vein (Cu) and splits into the media anterior (MA) ca. halfway through the wing length and the media posterior (MP) diverges from the cubitus near the distal third of the wing length; the cubitus vein runs along the wing margin until it splits into the cubitus anterior (CuA) and cubitus posterior (CuP) near the distal quarter of the wing; and a first anal vein fuses with the cubitus early on (Fig. 4B). Male tegmina have a simple subcoastal vein; a radial vein (R) which runs parallel with the media (M) throughout the full length of the wing and branches into the first radial (R1) ca. 2/5 of the way through the wing length and the primary vein terminates as the radial sector (Rs) at the wing apex; the media runs parallel with the radius and splits into the media posterior (MP) near the distal 2/5 of the wing, bends immediately and runs parallel with the media anterior (MA) until reaching the wing margin; the cubitus is unbranched and runs along the wing margin; and there is a first anal which fuses with the cubitus ca. ⅓ of the way through the wing length (Fig. 3B). Male alae (Fig. 3B) have a costal vein (C) running along the anterior margin and a subcostal vein (Sc) which runs for nearly the same length parallel with the costal vein; the radial vein (R) is bifurcate when it splits slightly less than 1/3 of the way through the wing length where they gradually diverge, run parallel/subparallel, then converge gradually to the apex where they terminating very near each other but not touching; the media (M) is also bifurcate, splitting about 1/8 of the way through the alae length and these veins run parallel for most of their length until the media posterior (MP) fades near the wing apex and the media anterior (MA) fuses with the radial sector near the wing apex; the cubitus (Cu) is fused with the first anterior anal (1AA) for ca. ¾ of the length until the first anterior anal splits and runs to the wing margin and the cubitus runs singularly to the wing apex; the anal veins are split into two groups, seven anterior anals (1AA–7AA) and five posterior anals (1PA–5PA).

***Abdomen*.** Abdominal shapes are variable within this genus; females are only known for *Vaabonbonphylliumgroesseri* (Zompro, 1998), comb. nov. and have a boxy abdomen (with segments IV through VI parallel sided) and segment VII strongly lobed and tapering strongly to a narrower segment VIII which is weakly lobed (Fig. 10B). For the males, *Vaabonbonphylliumgroesseri* comb. nov. have segment II converging to the narrowest point of the abdomen, III diverging (giving the abdomen a pinched waist appearance), IV weakly projecting and converging, V diverging, VI strongly diverging and converging giving it a notable lobe, VII appears variable as one male known has this segment converging (Fig. 11A) and the other male has a notable lobe on this segment as well (Fig. 11D), the remainder of the segments converge to the apex. In *Vaabonbonphylliumrafidahae* gen. et sp. nov. the abdominal shape is simple with all segments similar in width and margins all nearly parallel sided giving it a narrow, blade-like appearance (Fig. 12B).

Female subgenital plate is moderate in length and width, projecting slightly less than halfway under the terminal abdominal segment; gonapophyses VIII are long, broad, and parallel sided for most of their length, with an apex which projects from under the terminal abdominal segment about as much as the cerci do; the cerci are flat and marked sparsely with a granular surface (Fig. 10E). Males have an approximately equilateral triangular vomer which is singularly pronged, hooking up into the paraproct (Figs 11G, 12A).

***Egg.*** Egg morphology is not yet known for this rare genus.

###### Etymology.

*Vaabonbonphyllium* meaning “leaf that waits for the night to come”. This generic epithet is a compound of the Latinized name *Phyllium* the type genus for the family (from Greek φυλλον, -ου (*phyllon*, -*oy*) + -um; Poitout 2007), coupled with the prefix *vaabonbon* from the Teop (Tiop) language phrase which means “wait for the night to come” (Mosel 2019). We wished to honor the original inhabitants of this area by using a traditional language from Bougainville Island, the type locality of the genus. The Teop language is little known and considered a threatened language with as few as 6,000 speakers left in the autonomous region of Bougainville Island (Mosel 2014). We chose this name as these insects are exceptionally camouflaged and nocturnal, holding still during the day, thus hiding from view and only venturing out at night, making them exceptionally rare in collections and little known. This new genus is neuter in gender, following *Phyllium*.

###### Distribution.

At present *Vaabonbonphyllium* gen. nov. is primarily known from the Solomon Islands (*Vaabonbonphylliumgroesseri* comb. nov.) and a single record from Western Highlands Province, Papua New Guinea (*Vaabonbonphylliumrafidahae* gen. et sp. nov.; Fig. 5).

### ﻿New combination and new species

***Vaabonbonphylliumgroesseri* (Zompro, 1998), comb. nov.**

#### *Vaabonbonphylliumrafidahae* gen. et sp. nov.

##### ﻿ Vaabonbonphyllium groesseri

Taxon classificationAnimaliaPhasmatodeaPhylliidae

(Zompro, 1998) comb. nov.

EEB2E022-BC32-5E57-8BA0-D05782B22A9B

Figs 4B , 10 , 11

###### Material examined.

(15 ♀♀, 2 ♂♂, 3 ♀♀ nymph, 2 ♂♂ nymph, 2 unsexed nymph): ***Holotype*** (♀): “Buin I. Bougainville Arch. Salomons; 1941 4; *Chitoniscuslobiventris* Blanch. K. Gunther det.; *lobiventris* Blanch.; *Chitoniscus* Stal; *Phylliumgroesseri* Zompro 1997 HOLOTYPUS det. O. Zompro X. 1996; HOLOTYPUS; *Phylliumgroesseri* ♀ Zompro n. sp. HOLOTYPUS det. O.Zompro V.1998” (SMTD; Fig. 10A). See Suppl. material 1 for additional specimens reviewed, their collection data, and depositories.

###### Remarks.

At present this is the only known phylliid species from the Solomon Islands, although the phylliid knowledge of these islands is rather limited as even this species is rarely collected so if additional species were present but not yet formally described it would not be surprising.

Only two males are known to us at the present (from within the NHMUK and CSIRO collections) both of which are notably damaged (Fig. 11). These males have not yet been positively confirmed as the male *Vaabonbonphylliumgroesseri* comb. nov. via molecular comparison or from captive breeding but based upon shared morphology between the sexes and the current knowledge that only one species of phylliid is present on the Solomon Islands it is safe to assume these are the male for this species.

This species was originally described simply as a *Phyllium* species (not placed within a subgenus) and was placed within the Pulchriphyllium subgenus by Größer (2001) due to the presence of exterior lobes of the tibiae. During the intrageneric revision of Hennemann et al. (2009) this species was placed within the *frondosum* species group to which this species has a strong general resemblance (when reviewing the size, abdominal shape, and female wing venation). In 2020 the *frondosum* species group was found to represent the unknown female *Nanophyllium*; however, this species was not transferred to the *Nanophyllium* but was instead moved to the Pulchriphyllium subgenus schultzei species group based upon the bilobed protibiae (Cumming et al. 2020a). Now thanks to a full review of morphology and review of multiple specimens, the fine details which differentiate this clade from others allows its formal recognition as a unique genus.

###### Differentiation.

Female *Vaabonbonphylliumgroesseri* comb. nov. are the only females known for this genus, so differentiation from congenerics is not possible until the unknown female *Vaabonbonphylliumrafidahae* gen. et sp. nov. is located and described. Male *Vaabonbonphylliumgroesseri* comb. nov. can be differentiated from *Vaabonbonphylliumrafidahae* gen. et sp. nov. based upon the abdominal shape as *Vaabonbonphylliumgroesseri* comb. nov. has notably differing abdominal segment widths, with the middle segments many times wider than the anterior and distal segments (Fig. 11A, D) versus *Vaabonbonphylliumrafidahae* gen. et sp. nov. which has an abdomen with all segments relatively even in width giving it a smooth margined appearance (Fig. 12B). Also, the lobes of the pro- and mesofemora allow differentiation as *Vaabonbonphylliumgroesseri* comb. nov. has the profemoral exterior lobe highly reduced (Fig. 11B) and the interior mesofemoral lobe is notably broader than the mesofemoral exterior lobe versus *Vaabonbonphylliumrafidahae* gen. et sp. nov. which has a profemoral exterior lobe which is slightly wider than the profemoral shaft width (Fig. 12C) and the mesofemoral interior and exterior lobes which are approximately the same width (Fig. 12E). Additionally, the prosternum allows for differentiation as in *Vaabonbonphylliumgroesseri* comb. nov. the prosternum is relatively smooth, lacking a distinct protrusion (Fig. 11E) versus *Vaabonbonphylliumrafidahae* gen. et sp. nov. which has a distinct protrusion with a granular surface (Fig. 12H).

###### Distribution.

This species was originally only recorded from Bougainville Island, but since then additional records have been recovered from numerous collections representing previously unknown localities (see Suppl. material 1 for a full list of known specimens and their data). Additional islands recorded are (north to south): Kolombangara, Nggela, Guadalcanal, and Small Malaita (Fig. 5). With how rarely this species is collected, and from the wide range that it can be found from throughout the Solomon Islands, it is likely that this species can be found on several of the other larger islands as well, but just has not been formally recorded yet.

##### ﻿ Vaabonbonphyllium rafidahae

Taxon classificationAnimaliaPhasmatodeaPhylliidae

gen. et sp. nov.

15C8A36A-DF62-5248-9931-1F51106587C0

https://zoobank.org/D8265C9B-18D2-4C41-9697-829CCD2A0655

Figs 3B , 12

###### Material examined.

***Holotype*** ♂: “Papua New Guinea: Western Highlands Province (Highlands Region). Mt. Hagen Dist. (New Guinea Island) Mt. Hagen, along Highlands Hwy. (5 51'46.20"S, 144 13'30.75"E) Elev. 1730 m 23-VI-1989 Coll. L D. Munsey. (Coll RC 19-106)”. Deposited in the Montreal Insectarium (IMQC).

###### Remarks.

This mainland New Guinea species is presently only known from the singular holotype male (Fig. 12). This species is tentatively placed within *Vaabonbonphyllium* gen. nov. due to the protibial exterior being bilobed (a feature only known from *Vaabonbonphyllium* gen. nov. and *Rakaphyllium* gen. nov.) but the wing venation of this male differs notably from *Rakaphyllium* gen. nov. and therefore, this species appears to be related to *Vaabonbonphylliumgroesseri* comb. nov. based upon our poor knowledge of male morphology within that species. Unfortunately, for the *Vaabonbonphyllium* gen. nov. only three males are known (two *Vaabonbonphylliumgroesseri* comb. nov. and this herein described species) so our knowledge on the intrageneric variability is severely limited and until specimens of both species can be sequenced a confident placement cannot be made beyond this initial morphology-based review.

###### Differentiation.

Females unknown. Male *Vaabonbonphylliumrafidahae* gen. et sp. nov. can be differentiated from *Vaabonbonphylliumgroesseri* comb. nov. based upon the abdominal shape as *Vaabonbonphylliumrafidahae* gen. et sp. nov. has an abdomen with all segments relatively even in width giving it a smooth margined appearance (Fig. 12B), versus *Vaabonbonphylliumgroesseri* comb. nov. which have notably differing abdominal segment widths, with the middle segments many times wider than the anterior and distal segments (Fig. 11A, D). The lobes of the femora allow differentiation as *Vaabonbonphylliumrafidahae* gen. et sp. nov. has a profemoral exterior lobe which is slightly wider than the profemoral shaft width (Fig. 12C) and mesofemoral interior and exterior lobes which are approximately the same width (Fig. 12D) versus *Vaabonbonphylliumgroesseri* comb. nov. which has the profemoral exterior lobe highly reduced (Fig. 11B) and the interior mesofemoral lobe notably broader than the mesofemoral exterior lobe. Additionally, the prosternum allows for differentiation as in *Vaabonbonphylliumrafidahae* gen. et sp. nov. there is a distinct protrusion (Fig. 12H), and in *Vaabonbonphylliumgroesseri* comb. nov. the prosternum is relatively smooth, lacking a distinct protrusion (Fig. 11E).

###### Distribution.

Currently only known from the type locality of Mt. Hagen, Western Highlands Province, Papua New Guinea (Fig. 5).

**Male. *Coloration*.** The coloration description is based on the single dried holotype specimen which appears to have been collected in alcohol which turns phylliids yellow (Fig. 12). In life the specimen likely was green, but color variation is known within phylliids so a definitive coloration description cannot be given at this time for possible living coloration. The overall coloration of the holotype specimen is straw yellow throughout with the only feature which is distinctly different in color being the compound eyes which are a dark red (Fig. 12G). Abdominal segment V has a set of eye spots which are ovular and slightly darker in color than the remainder of the abdomen.

***Morphology*. *Head*.** Head capsule approximately as long as wide, with a vertex that is marked throughout by nodes which are relatively evenly spaced and of uniform size (Fig. 12G). The posteromedial tubercle is singularly pointed, large, and notably raised above the head capsule, many times larger than any of the capsule granules (Fig. 12H). Frontal convexity stout and bluntly pointed with several short setae on the apex. Compound eyes large and bulbous, occupying ca. ⅖ of the head capsule lateral margins (Fig. 12G). Between and slightly posterior to the compound eyes are three ocelli that are well-developed (Fig. 12G). Antennal fields are slightly wider than and approximately as long as the scapus.

***Antennae*.** Antennae (including the scapus and pedicellus) consist of 22 segments. The scapus and pedicellus are bare, segments III through XIX have dark setae which are mostly around two times longer than the segments are wide and are not densely packed, the terminal three segments are covered by fine, densely packed, transparent setae (Fig. 12F).

***Thorax*.** Pronotum is slightly longer than the greatest width, with anterior margin concave and lateral margins that are straight and converge to a gently convex posterior margin that is ca. ⅗ the width of the anterior rim (Fig. 12G). Anterior and lateral margins of the pronotum have distinct rims and the posterior margin lacks a rim (Fig. 12G). Face of the pronotum is notably lumpy, marked with a distinct sagittal furrow on the anterior half, and a distinct pit in the center (Fig. 12G). Prosternum has a notably granulose surface with a distinctly projecting area in the center and an additional smaller projection near the posterior margin, which is smaller than the central projection, both projections are marked with granulation (Fig. 12H). Mesosternum has a notably granulose surface similarly marked as the prosternum with uniform spacing between granules which are all nearly even in size. Metasternum not as heavily marked with granulation, but instead has slightly wrinkled anterior areas and a nearly smooth central and posterior area. Prescutum on the anterior is wider than long, with lateral margins converging uniformly to the posterior which is ca. ⅗ as wide as the anterior rim width (Fig. 12G). Lateral rims with a lumpy texture due to irregularly sized tubercles/nodes (ca. nine distinct but some weakly formed) with the largest near the anterior margin (Fig. 12G). The surface of the prescutum is marked with a few nodes but it is mostly smooth as there is no distinct sagittal crest, instead the anterior margin is the only distinct feature as the rim is prominently formed with a granular surface and raised into a distinct sagittal point (Fig. 12G, H). Mesopleura narrow, only slightly diverging from the anterior to the posterior giving the mesosternum a rectangular appearance when viewed ventrally. Lateral margin with one notably larger tubercle on the anterior margin with three setae protruding from it followed by four or five moderately sized tubercles which in some cases have a seta or two protruding from them, with these tubercles evenly spaced throughout the length (Fig. 12G). Face of the mesopleura with a weakly lumpy surface (most areas are relatively smooth) and marked with two weakly formed pits near the middle of the length (Fig. 12G, H).

***Wings*.** Tegmina moderate length, extending ca. ⅓ of the way onto abdominal segment III (Fig. 3B). Tegmina wing venation: the subcosta (Sc) is the first vein, it runs relatively straight and terminates slightly less than ½ of the way through the overall tegmina length. The radius (R) spans the entire length of the tegmina with the first radius (R1) branching slightly greater than ⅓ of the way through the wing length and running to the wing margin where it terminates ca. ⅗ of the way through the wing length, and then the radial sector (Rs) runs straight from the branching point to the wing apex. The media (M) spans the entire length of the tegmina, terminating at the wing apex as the media anterior (MA). The media posterior (MP) branches ca. ⅔ of the way through the wing length, immediately bends and runs parallel with the media anterior until it terminates near the wing apex. The cubitus (Cu) runs through the wing surface angled towards the margin which it meets ca. ⅓ of the way through the tegmina length at the same location where the first anal (1A) vein fuses with it and the cubitus runs along the wing margin until it fades near the point where the media posterior terminates near the wing margin. Alae well-developed in an oval fan configuration, reaching beyond the apex of the abdominal segments, instead reaching to the apex of the cerci. Alae wing venation (Fig. 3B): the costa (C) is present along the entire forewing margin. The subcosta (Sc) runs along the costa throughout almost its entire length. The radius (R) branches ca. ¼ of the way through the wing length into the first radius (R1) and radial sector (Rs) which run for half of their length slightly diverging, then parallel/subparallel until the wing apex where thy converge and terminate very near each other at the wing apex. The media (M) branches early, ca. ⅛ of the way through the wing length into the media anterior (MA) and the media posterior (MP) which run parallel with each other until the media anterior bends towards the radial sector and fuses with it near the wing apex, and the media posterior bends towards the media anterior but fades before fusing with it. The cubitus (Cu) runs unbranched and terminates at the wing apex. Of the anterior anal veins, the first anterior anal (1AA) fuses with the cubitus near the point where the media branches into the media anterior and media posterior and then the first anterior anal branches from the cubitus ca. ¼ of the way through the wing length where it uniformly diverges from the cubitus until it terminates at the wing margin. The anterior anal veins two–seven (2AA–7AA) have a common origin and run unbranched in a folding fan pattern of relatively uniform spacing to the wing margin. The posterior anal veins (1PA–5PA) share a common origin separate from the anterior anal veins and run unbranched to the wing margin.

***Abdomen*.** The general shape of the abdomen is long and slender with margins that are relatively parallel and segments which are nearly even in width, varying little from one to the next (Fig. 12B). Abdominal segments II through IV diverge only slightly, segments V and VI are parallel sided, and VII through the apex converge to the apex.

***Genitalia*.** Poculum roundly rectangular in shape with anterior and lateral margins relatively straight and the posterior margin only slightly passing onto the terminal abdominal segment as it gently arcs towards the abdomen apex. Cerci long and slender (approximately ⅖ as wide as long), with slightly more than ½ of their length extending out from under the anal abdominal segment. The cerci are relatively flat and covered in a granulose surface with numerous short setae evenly spaced throughout (Fig. 12A). The vomer general shape is an equilateral triangle with straight sides evenly converging to the apex, which is armed with a singular upward turning hook that is broad and notably darker than the rest of the vomer (Fig. 12A).

***Legs*.** Profemoral exterior lobe arcing gently with a width only slightly greater than the greatest width of the profemoral shaft with the margin marked slightly with granulation (Fig. 12D). Profemoral interior lobe roundly right angled with a greatest width ca. three times that of the exterior lobe and the distal margin is marked with three or four stout and blunted teeth with varying spacing between them (Fig. 12D). Mesofemoral exterior lobe arcs unevenly end to end with the widest point on the distal ⅓, and the greatest width slightly wider than the mesofemoral shaft or mesofemoral exterior lobe widths (Fig. 12D). The mesofemoral interior lobe is marked by four dulled teeth on the distal ⅓ of the lobe (Fig. 12D). The mesofemoral exterior lobe is very similar to the interior lobe in terms of shape, size, and dentition, with the only notable difference being that at its greatest width it is slightly thinner than the interior lobe (Fig. 12D). Metafemoral exterior lobe has a slightly granular margin but lacks dentition, and the margin is straight running along the metafemoral shaft (Fig. 12E). Metafemoral interior lobe is unevenly weighted on the shaft with the majority occupying the distal ⅔ of the length with the distal ⅓ marked with three or four teeth, with the proximal most rather dulled and the distal most teeth more finely pointed (Fig. 12E). Protibiae with two exterior lobes, one small lobe on the distal end and a larger lobe (ca. three times as large as the small distal lobe) slightly proximal to halfway through the length (Fig. 12D). Protibiae with a single interior lobe which is the shape of a rounded scalene triangle which occupies the proximal ¾ of the protibial length and at its maximum width is ca. two and a half times the width of the protibial shaft width (Fig. 12D). Mesotibiae and metatibiae lack interior lobes but both have singular small exterior lobes situated on the distal end, with the mesotibial exterior lobe only weakly formed (not as wide as the shaft width: Fig. 12D) and the metatibial exterior lobe more distinctly formed (ca. as wide as the metatibial shaft width: Fig. 12E).

###### Measurements of holotype male [mm].

Length of body (including cerci and head, excluding antennae) 52.8, length/width of head 3.1/3.0, antennae 21.5, pronotum 2.5, mesonotum 2.3, length of tegmina 17.2, length of alae 45.6, greatest width of abdomen 10.5, profemora 7.7, mesofemora 7.4, metafemora 7.4, protibiae 4.8, mesotibiae 4.8, metatibiae 6.8.

###### Etymology.

The specific epithet of the new species is selected to honor the wife of Larry D. Munsey, the individual who collected the specimen upon which the new species description is based. Rafidah, a native of West Malaysia, and Larry, a native Californian, live in Borneo where they jointly have been studying the megadiverse cerambycid fauna of the island for the past 17 years.

#### Phylliinae Brunner von Wattenwyl, 1893

**Nanophylliini Zompro & Größer, 2003**

##### ﻿ Acentetaphyllium gen. nov.

Taxon classificationAnimaliaPhasmatodeaPhylliidae

FFFBB863-2DB2-5389-ADE7-9721770F577A

https://zoobank.org/A5CCCEC7-1112-4961-A9EA-503B393B8CFB

###### Type species.

*Phylliumbrevipennis* Größer, 1992: 164, herein designated.

###### Taxonomic hierarchy.

This clade, based upon molecular analyses, was recovered as sister to the *Nanophyllium* a placement which is supported by the linked morphological features of the wider than long prescutum, two lobed posteromedial tubercle, and similar antennae morphology (Bank et al. 2021). Therefore, this new genus is included within the tribe Nanophylliini Zompro & Größer, 2003 along with its sister genus *Nanophyllium*.

###### Discussion.

The selected type species for this new genus is *Phylliumbrevipennis* Größer, 1992 (= *Acentetaphylliumbrevipenne* (Größer, 1992), comb. nov.) as this was the first species described from this group and is represented by a good condition holotype female with detailed collection data so little confusion surrounds the type species. Additionally, this species has been sequenced and included in molecular phylogenies (Bank et al. 2021) while the other species are still lacking.

Again, the uniqueness of this clade was recognized by Hennemann et al. (2009), with their designation of the *brevipenne* species group, and they even recognized this clade as closely related to the *Nanophyllium* (which as the time were still considered the Phyllium (Pulchriphyllium) frondosum species group, not the opposite sex of the *Nanophyllium* yet which would not be until a decade later in Cumming et al. 2020a).

**Figure 13.**
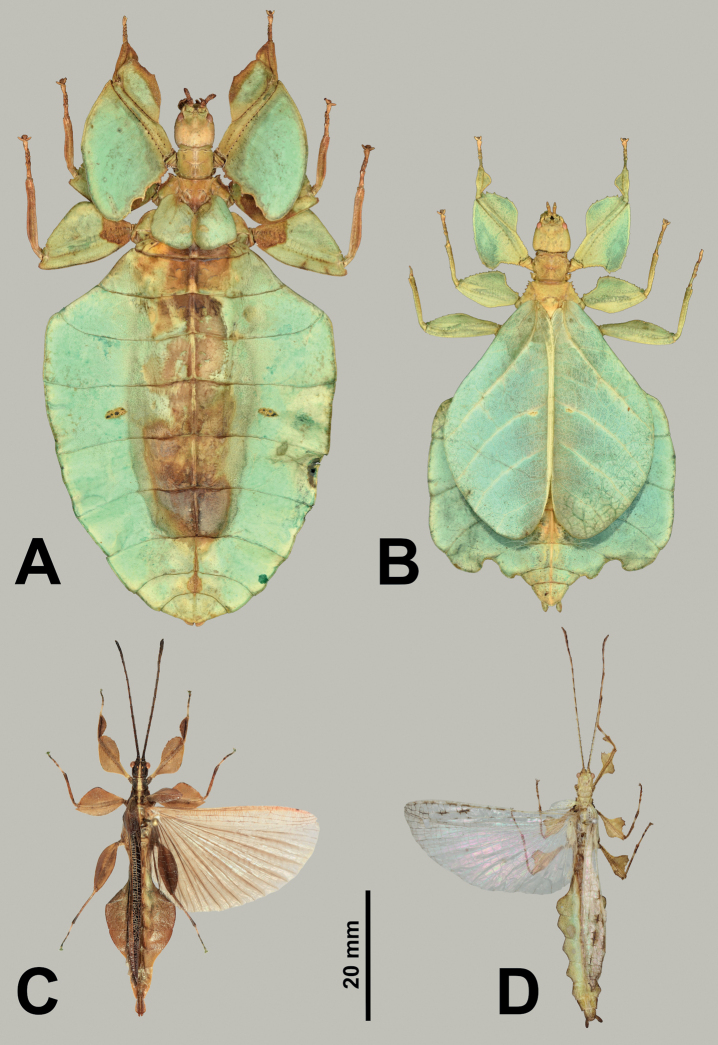
Dorsal habitus of adult *Acentetaphyllium* gen. nov. and *Nanophyllium* to help illustrate the morphological differences/similarities between these two genera. Images scaled to relative size; scale bar associated with all images **A***Acentetaphylliumbrevipenne* female (Coll TM) **B***Nanophylliumchitoniscoides* (Größer, 1992) female (Coll TM) **C***Acentetaphylliumstellae* comb. nov. holotype male (SDNHM) **D***Nanophylliumrentzi* holotype male (NHMUK) **A, B** photographs by Rene Limoges (IMQC) **C** photograph by first author **D** photograph by Paul Brock (NHMUK).

Within the phylliid-wide phylogeny of Bank et al. (2021)*Acentetaphylliumbrevipenne* comb. nov. was recovered as sister to all sampled *Nanophyllium* species with high support. Despite the drastic morphological differences between this new genus (Fig. 13A) and the *Nanophyllium* sensu stricto (Fig. 13B), there are still several morphological features which help to link these two genera. Most notably the two lobed posteromedial tubercle of the head capsule and the similar mesothorax morphology, which is notably wider than long, immediately link these two genera.

**Figure 14.**
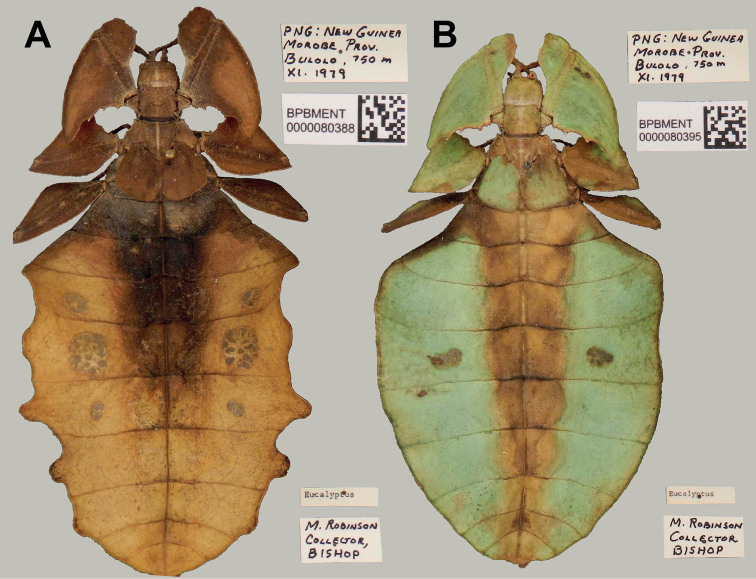
Examples of abdominal variation observed within *Acentetaphylliumbrevipenne* comb. nov. adult females from within the BPBM collection, dorsal habitus, with their associated data labels to the right of each specimen. Photographs by Miho Maeda, Jerilynn Chun, and James Boone, 2020 (BPBM) **A** brown form with distinct eye spots on three of the abdominal segments, and a notably lobed abdomen **B** green form female with eye spots on a single abdominal segment and an abdominal shape which has smoother margins.

Little is known about the *Acentetaphyllium* gen. nov. ecology except for labels included with two females from within the BPBM collection noting their host plant as “Eucalyptus” (Fig. 14). Several other genera/species are known to accept *Eucalyptus* spp. as a host within captivity (pers. comm. Tomas Stijnts (Belgium; Flanders)). Additionally, these BPBM specimens also note an elevation of collection at 750 meters, whereas a specimen in the Coll SLT from Wau, Morobe Province was collected at 1,200 meters, suggesting this species is not restricted to a limited elevation range.

Within the *Nanophyllium* the association of opposite sexes has always been problematic due to limited material and notable age of many specimens not allowing molecular comparison. Only recently was the confusion surrounding the “missing” *Nanophyllium* females for the most part been resolved (Cumming et al. 2020a). However, even within that work it was recognized that there were two distinct clades known for the males (noted within Cumming et al. 2020a as the *pygmaeum* species group and the *stellae* species group, see therein figures and side-by-side morphological comparisons) but females were only confirmed for the *pygmaeum* species group. The *stellae* species group was recognized as having numerous morphological similarities and differences to the *pygmaeum* species group but no tentative female had been linked with clarity. Now with the molecular results of Bank et al. (2021) recovering *brevipenne* as sister to the *Nanophyllium*, the males of the *stellae* species group can with higher confidence be associated with these morphologically unique females (see Fig. 13 for a side by side of these two clades). Although not yet confirmed by breeding or from molecular comparison, the numerous shared morphological features of the males of the *stellae* species group and *brevipenne* strongly suggest these are opposite sexes of the same clade (Fig. 13A, C).

For detailed discussion and figures of the non “*brevipenne*” *Acentetaphyllium* gen. nov. species which were originally placed within *Nanophyllium*, see the recent work of Cumming et al. (2020a) as these species (*Acentetaphylliumlarssoni* (Cumming, 2017), comb. nov.; *Acentetaphylliummiyashitai* (Cumming et al., 2020), comb. nov.; and *Acentetaphylliumstellae* (Cumming, 2016), comb. nov.) are not discussed below as no additional information can be added at this time to supplement the recent publication and their original descriptions.

###### Morphological differentiation from *Nanophylliumsensu stricto*.

**Figure 15.**
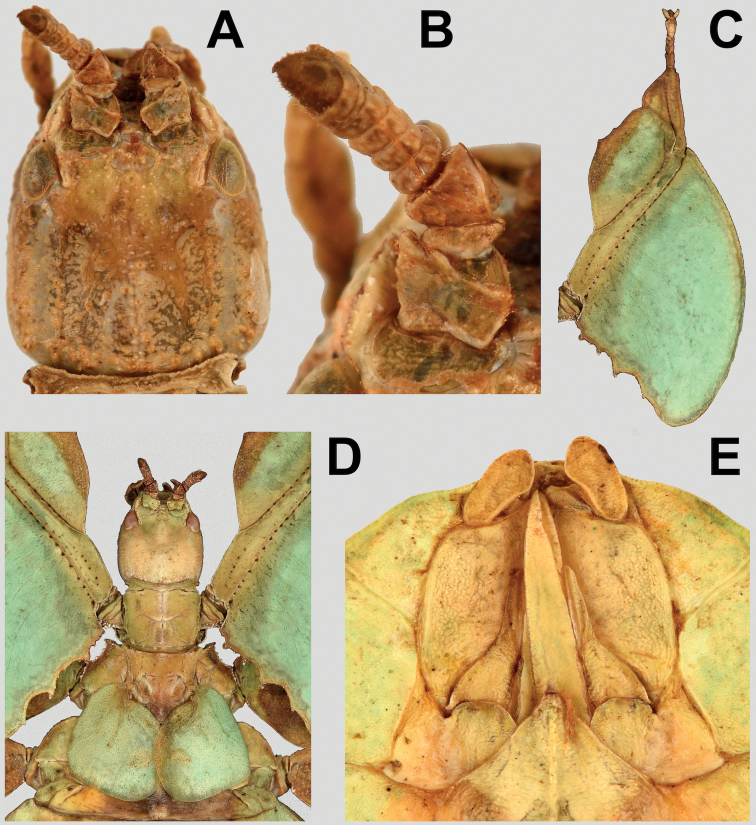
*Acentetaphylliumbrevipenne* comb. nov. adult female details **A, B, E** from Coll SLT**C, D** from Coll TM Photographs by Rene Limoges (IMQC) **A** details of head, dorsal **B** details of antennae, dorsal **C** details of protibiae and profemora, dorsal **D** details of head through thorax, dorsal **E** details of genitalia, ventral.

Features which link these two genera are their antennae morphology (in females), thorax shape and spination (both sexes), and genitalia (both sexes). There are several notable differences between the genera, for female *Acentetaphyllium* gen. nov. the tegmina are highly sclerotized and rudimentary (Fig. 15D), at most reaching onto the anterior of abdominal segment II leaving the dorsal of the abdomen fully exposed versus tegmina which are fully developed and covering most of the abdomen in *Nanophyllium*. For male *Acentetaphyllium* gen. nov. the shape of the femoral lobes differentiates them as the profemoral interior lobes are rounded without a sharp angle and the mesofemoral interior lobes have a large rounded triangular lobe, reaching from end to end and lacking prominent spination (versus *Nanophyllium* which have profemoral interior lobes which are angular and mesofemoral interior lobes which are heavily weighted towards the distal end and are marked by distinct serrate teeth on the distal half). Also, the alae of the male *Acentetaphyllium* gen. nov. allow differentiation as the media anterior and the media posterior do not fuse, instead they both run to the wing margin, and the cubitus after splitting from the first anterior anal fuses with the media posterior near the wing margin and they run fused to the wing apex (versus *Nanophyllium* which have the media posterior fusing to the media anterior and then they run fused to the wing margin without fusing with other veins (see Cumming et al. 2020a: figs. 10–13 for side by side comparisons)).

###### Autapomorphic features.

For females the tegmina are highly sclerotized and rudimentary, no longer than the anterior of abdominal segment II (Fig. 14), whereas all other phylliids have well-developed tegmina which at least reach halfway onto the abdomen and in most cases cover most of the abdomen. For males the alae venation is autapomorphic as the media anterior and the media posterior do not fuse, instead the media anterior runs alone to the wing margin and the cubitus (after splitting from the first anterior anal) fuses with the media posterior near the wing margin and then they run fused to the wing apex as one (see Cumming et al. 2020a: fig. 13B for an illustration of this venation pattern).

###### Generic characteristics.

The *Acentetaphyllium* gen. nov. have females that for phylliids are average length (ca. 80 mm) and males which are on the smaller end (ca. 40 mm), which gives a male to female length ratio of ca. 1.0:2.0 between the sexes for this genus which is the most extreme known in the family (Fig. 13A, C) as typically a male to female ratio of 1.0:1.2 to 1.0:1.6 is common. Interestingly for *Acentetaphyllium* gen. nov. coloration, males are only known to have brown forms but within females brown, green, and yellow are all known to exist. Hopefully as more males are located it will become apparent if males are exclusively brown or also come in different color forms.

***Antennae*.** Females have antennae with nine segments with segments IV through VIII which are uniform in length, disk-like, marked with granulation, and the terminal segment is stout and densely covered in setae (Fig. 15B). Males have antennae with 21 to 22 segments which are relatively flat, and most segments are covered by setae which are longer than the segment is wide.

***Head capsule*.** Males have well-developed ocelli and females lack ocelli. Males have compound eyes which are strongly protruding and occupy ca. 2/5 of the head capsule lateral margins versus females which have compound eyes which are notably smaller, only occupying ca. ¼ of the head capsule lateral margins and which to not strongly protrude from the capsule (Fig. 15A). Both sexes have head capsules which are marked throughout by distinct but somewhat irregularly sized granulation which is relatively evenly spaced (Fig. 15A).

***Thorax*.** In both sexes the prescutum can be two to three times wider on the anterior than long with lateral margins that are granular, and a prescutum surface which is only slightly granular, lacking a prominent anterior rim or sagittal crest (Fig. 15D). In both sexes the mesopleurae begin notably wider than the prescutum anterior rim and weakly diverge gradually and can be marked with a single anterior tubercle or by as many as nine warty tubercles (Fig. 15D).

***Legs*.** Both sexes have interior protibial lobes which span the full length in a broadly rounded triangle, lack lobes on the protibial exterior, and the meso-, metatibiae are simple, lacking both interior and exterior lobes. In both sexes the profemoral interior lobe is notably smaller than the exterior lobe and is rounded without a strong angle and marked by two to four dulled weakly formed teeth (Fig. 15C). The profemoral exterior lobe is somewhat variable in males (being roundly obtuse or with a slight recurve giving the lobe a boxier appearance) but in females it appears to be more stable as it is notably recurving posteriorly and marked with a strongly serrate posterior margin and a smoothly arcing anterior margin (Fig. 15C). Both sexes have the interior meso-, and metafemoral lobes ca. 1.5 to 2.0 times as broad as the exterior lobes, with both arcing smoothly, giving the meso-, and metafemora smooth, pointed leaflet-like appearances which tuck in nicely to the anterior of the abdomen helping to give the habitus a full ovoid appearance (Fig. 14).

***Wings*.** In both sexes the tegmina are highly reduced and heavily sclerotized, with a maximum length of reaching the anterior margin of abdominal segment II, but mostly shorter, only reaching the metanotum (Fig. 15D). Females lack alae but in males the alae are fully developed, reaching onto abdominal segment IX or X. Male alae have a costal vein running along the anterior margin; a subcostal vein which runs alongside the costal vein for most of its length until it fuses with the costal vein near the wing margin; the radial vein is bifurcate, splitting into the first radial and radial sector near the distal third of the alae and these run to the wing margin without fusing to other veins or each other; the media splits early on into the media anterior and media posterior which run parallel to the wing apex, with the media anterior arriving alone, but the media posterior has the cubitus fuse to it near the distal 1/8 of the wing apex; the cubitus is fused with the first anterior anal for ca. half of the wing length until the first anterior anal splits and runs to the wing margin and the cubitus fuses with the media posterior; the anal veins are split into two groups, the anterior anals and the posterior anals (with seven anterior anals and six posterior anals).

***Abdomen*.** Abdominal shapes are somewhat variable; females are often perfectly spade-shaped (Fig. 14B) but in some forms the abdominal segments can have slightly undulating margins, giving them a wavy appearance (Fig. 14A); males have similar appearances as they can be smoothly spade-shaped (Fig. 14C) or can have abdomen which are strongly undulating (see *Acentetaphylliumlarssoni* comb. nov., for an example of a strongly undulating abdomen in males). Female subgenital plate is short and stout with the apex only reaching halfway onto abdominal segment IX and ending in a fine point; gonapophyses VIII are long and slender, but not quite reaching to the apex of the terminal abdominal segment; gonapophyses IX are located to either side of the gonapophyses VIII and have broad bases for ca. ½ their length which taper quickly to slender points; the cerci are relatively flat and rounded, with a slightly wrinkled surface (Fig. 15E). Males have a narrow, triangular vomer which is ca. two times longer than the greatest width and terminates in a single hook. Male cerci are notably marked with granulation and distinct setae along the margins.

***Egg.*** Egg morphology is not yet known for this rare genus.

###### Etymology.

*Acentetaphyllium* meaning “flawless leaf”. This generic epithet is a compound of the Latinized name *Phyllium* the type genus for the family (from Greek φυλλον, -ου (*phyllon*, -*oy*) + -um; Poitout 2007), coupled with the Latin prefix *acenteta* meaning “flawless”. One of the most remarkable features of this genus is how the abdomen and legs are shaped so that when the legs are at rest the habitus is a perfect oval, giving this genus a flawless leaf-like shape (Fig. 14B). This new genus is neuter in gender, following *Phyllium*.

###### Distribution.

At present only known from a handful of records as this appears to be a rarely collected genus. However, it appears as though this genus is found throughout the island of New Guinea as an undescribed *Acentetaphyllium* gen. nov. is known to us from Fak Fak, Indonesia, there are multiple records from northern New Guinea from the Cyclops Mountains (Indonesia side) and Bewani Mountains (Papua New Guinea side), and multiple records from Morobe Province, Papua New Guinea. See Suppl. material 1 for details on known records.

### ﻿New combination

***Acentetaphylliumbrevipenne* (Größer, 1992), comb. nov.**,

***Acentetaphylliumlarssoni* (Cumming, 2017), comb. nov.**,

***Acentetaphylliummiyashitai* (Cumming et al., 2020), comb. nov.**,

***Acentetaphylliumstellae* (Cumming, 2016), comb. nov.**

## ﻿Discussion

### ﻿The genus concept within Phylliidae

Due to a lack of scientific objectivity for determination of taxonomic ranks, taxonomic levels are inherently arbitrary, opening up a great deal of personal preference by the participating taxonomists (de Queiroz and Gauthier 1992). In many cases it is likely the historical precedent set forth by past taxonomists which have persisted through the ages that have defined the various taxonomic rankings that are arbitrarily yet habitually followed today across the plethora of organisms and their respective scholars. Therefore, the results of taxonomic rank determinations vary with author preference and often across organismal groups.

To be explicit with our taxonomic intentions, the goal of the current authors is for the phylliid taxonomy to: 1) reflect our understanding of their recovered phylogenetics, 2) that the arbitrary taxonomic ranks be assigned to monophyletic clades, and 3) that these ranks carry with them meaningful differentiation points for communications sake. This of course results in some taxonomic ranks having different degrees of differentiation (such as inferred clade age or degree of species diversity within a clade). Therefore, sometimes different clades assigned to the same taxonomic rank may not be perfectly comparable in a phylogenetic sense. Even though taxonomic ranks are arbitrary, our goal with adjusting the supraspecific phylliid taxonomy is to reflect natural groupings which carry with them human interpretable (and therefore useful) meaning.

For example, if someone were to discuss a male *Acentetaphyllium* one would immediately know they were referencing a male that morphologically the alae have the media anterior which runs alone to the alae apex, that the media posterior fuses with the cubitus and these fused veins run to the apex as MP+Cu, and phylogenetically that this specimen falls within a clade that is molecularly sister to the *Nanophyllium* yet morphologically distinct (as *Nanophyllium* males have alae where the media posterior fuses with the media anterior and this runs to the wing apex as MA+MP). Despite the arbitrary nature of the genus rank in this case, a great deal of pertinent information can easily be gleaned.

Alternatively, as taxonomic ranks are arbitrary, one could just as easily suggest making the *Nanophyllium* and *Acentetaphyllium* “subgenera”. This conundrum was explored within the supplementary materials of Bank et al. (2021) for how “genera” versus “subgenera” might be treated in the revision of paraphyletic clades. This issue was discussed because historically *Phyllium* Illiger, 1798 contained two subgenera which were just as morphologically and phylogenetically unique as other historic genera described around the same time. In light of the phylogenetic results recovering sister clades which were not nested within each other but instead were rather well-defined (and because the taxonomic rank of “subgenus” implies a certain degree of nestedness) instead it was decided that these easily morphologically differentiated groups be given the arbitrary rank of genus. As the phylliids currently stand (based upon precedent within the group set forth by past author preferences) significant wing venation differences coupled with notable phylogenetic distances often correspond to the artificial rank of genus. So, for simplicity’s sake, due to the significant wing venation differences and notable molecular distance between various clades such as the herein discussed *Nanophyllium* and *Acentetaphyllium* gen. nov. we are treating them as genera. Even though clades such as *Microphyllium*, *Pseudomicrophyllium*, *Nanophyllium*, and *Acentetaphyllium* gen. nov. are not evolutionarily as old as the *Chitoniscus* or *Walaphyllium*, all of these clades are monophyletic and carry with them associated morphological features which are easily observed. Thus, these taxonomic units carry with them certain diagnostic and useful information with them despite their arbitrary nature. To summarize the current treatment of genera within the Phylliidae, a summary table is presented to highlight key morphological features which allow differentiation of all phylliid genera (Table 1).

**Table 1.** Summary of key morphological features for differentiating phylliid genera and well as the clade geographic distribution.

Clade / Author	Geographic distribution	Males	Females
*Nanophyllium* Redtenbacher, 1906	Southern Indonesia to New Guinea and Northeastern Australia.	Alae, the media posterior fuses to the media anterior and then they run fused to the wing margin without fusing with other veins and bilobed posteriormedial tubercle of the head capsule.	Posteriormedial tubercle of the head capsule split into two lobes and tegmina well-developed, reaching onto abdominal segment VII or VIII.
*Acentetaphyllium* gen. nov.	New Guinea.	Bilobed posteriormedial tubercle of the head capsule and in the alae, the media anterior and the media posterior do not fuse, instead they both run to the wing margin, and the cubitus after splitting from the first anterior anal fuses with the media posterior near the wing margin and they run fused to the wing apex.	Bilobed posteriormedial tubercle of the head capsule and tegmina rudimentary, at most only reaching onto the anterior of abdominal segment II.
*Walaphyllium* Cumming et al., 2020	New Guinea and Northeastern Australia.	Abdominal shape rectangular, with segments V and VI with fully parallel-sided margins (segments IV and VII with only half parallel-sided and the remainder curved) and alae radial sector, media anterior, and media posterior not fusing with the cubitus; metafemora exterior simple, lacking a lobe.	Tegmina venation with the posterior cubitus split into an anterior cubitus (CuA), first posterior cubitus (CuP1), and second posterior cubitus (CuP2).
*Vaabonbonphyllium* gen. nov.	New Guinea and Solomon Islands.	Profemora exterior with two lobes, one near the middle of the length and one on the distal end and in the alae the radius is bifurcate.	Protibiae exterior with two lobes (one on the distal end and one closer to the proximal end) and gonapophyses VIII average in length, with a majority of their length under the terminal abdominal segment, only the tips project from the apex.
*Rakaphyllium* gen. nov.	New Guinea and Aru Islands.	Protibiae exterior with two lobes (one on the distal end and one closer to the proximal end) and alae, radius vein is simple, does not split.	Protibiae exterior with two lobes (one on the distal end and one closer to the proximal end) and gonapophyses VIII long, with ca. half of their length projecting from under the terminal abdominal segment.
*Pulchriphyllium* Griffini, 1898	Seychelles, India to mainland Asia, to western Indonesia islands.	Alae radial sector, media anterior, and media posterior veins fusing to the cubitus at different locations along the vein and running together to the wing margin and metafemora exterior with a prominent lobe spanning the full length of the shaft.	Pro-, meso-, and metatibiae exterior with lobes; and tegmina with media and cubitus veins running side by side, touching throughout most of their lengths.
*Pseudomicrophyllium* Cumming, 2017	Philippines (Luzon).	Antennae notably longer than the outstretched front legs, with antennomeres 4–5× longer than wide and profemoral interior lobes reduced to only a single anterior spine.	Posterior most spine of the prescutum is the most prominent, gonapophyses VIII which are long, reaching to the apex of the anal abdominal segment and third antennomere with stridulatory file.
*Microphyllium* Zompro, 2001	Philippines (Luzon).	Antennae short (only ca. the length of the outstretched front legs), with bead-like antennomeres that are no more than 2× longer than they are wide and profemoral interior lobes which are narrow and marked with three distinct teeth.	Middle most spine along the prescutum is the largest spine and the posterior most spine is highly reduced and third antennomere lacking stridulatory file.
*Comptaphyllium* Cumming et al., 2019	Southern Indonesia to New Guinea.	Tegmina media vein splits into the anterior media vein (MA) and posterior media vein (MP) very early on, immediately or at most 1/4 of the way through the wing length and they run unbranched and subparallel through the wing length; protibial interior lobe not reaching from end to end of the shaft, only restricted to the proximal 1⁄2 to 2⁄3 but never more; a head capsule with clearly defined nodes arranged in evenly spaced patterns.	Terminal antennomere as long as the preceding three to five segments combined; pprescutum sagittal plane either with: the spine on the anterior rim most prominent.
*Chitoniscus* Stål, 1875	Fiji.	Ocelli absent, alae media anterior (MA) runs unfused to the wing margin and media posterior (MP) fades without fusing or reaching the wing margin, and prescutum stout, ca. 2× wider than long.	Coxae ventrally are sky blue in color and prescutum anterior rim strongly protruding and distinctly angled posteriorly.
*Trolicaphyllium* Cumming et al. 2021	New Caledonia.	Ocelli well-developed and in the alae, media anterior (MA) and media posterior (MP) veins fuse with the cubitus (Cu) at different locations along the cubitus and run fused to the wing margin.	Coxae ventral color is the same color as the legs and thorax, prescutum anterior rim and spine not strongly protruding posteriorly, only slightly raised and vertical, and tegmina radial vein (R) runs parallel with media (M) until the split of the radial sector (Rs), at which point the radial sector (Rs) bends away distinctly.
*Cryptophyllium* Cumming et al., 2021	Mainland Asia to north/central Indonesia, Southern Philippines, and Micronesia.	Vomer with two apical hooks.	Third antennomere with the proximal end broad and often slightly recurved, making the segment a similar width throughout, or broader on the proximal end; fourth antennomere short and disk-like at least 3× wider than long and notably shorter than any of the following three segments, or rarely a similar length to the following segment, but still at least 2× as wide as long.
*Phyllium* Illiger, 1798	Malaysia, throughout Indonesia and Philippines, to New Guinea and offshore islands.	Tegmina media vein running unbranched for the first 1⁄3 to 2⁄5 of the wing length, and then branching with either a single short media posterior running to the wing margin or two short media posteriors branching from the notably longer media anterior and running to the margin; protibial interior lobe variable, either fully spanning the full length or only 1⁄2 of the length; head capsule at most with random granulation but frequently bare; Abdominal shape variable, either spade-shaped (with the margins of V parallel or strongly converging and segment VI strongly converging), ovular (with margins expanding and then contracting, no segments parallel-sided), thin and slender with converging margins, bell-shaped (with margins expanding until segment VI then strongly converging) or boxy with only segment V parallel-sided (segments IV and VI only partially parallel-sided, the remainder rounded	Third antennomere narrowest on the proximal end, broadening to the distal end; fourth antennomere typically as long as wide and of a similar length to each of the following three segments length, not notably shorter; tibiae lacking exterior lobes; many other features rather variable

### ﻿Keys to Phylliidae genera

These keys are adapted from Cumming et al. (2021b) and the supplementary materials of Bank et al. (2021). Unfortunately, the egg and freshly hatched nymph morphology is unknown for the three herein described genera, therefore only keys to adult females and males are revised.

### ﻿Key to females

**Table:** 

1	Posteriormedial tubercle of the head capsule split into two lobes	**2**
–	Posteriormedial tubercle of the head capsule singular, not split into two lobes	**3**
2	Tegmina well-developed, reaching onto abdominal segment VII or VIII	***Nanophyllium* Redtenbacher, 1906**
–	Tegmina rudimentary, at most only reaching onto the anterior of abdominal segment II	***Acentetaphyllium* gen. nov.**
3	Tegmina venation with the posterior cubitus split into an anterior cubitus (CuA), first posterior cubitus (CuP1), and second posterior cubitus (CuP2)	***Walaphyllium* Cumming et al., 2020b**
–	Tegmina cubitus venation simple (unsplit) or bifurcate (into an anterior cubitus (CuA) and posterior cubitus (CuP1) only)	**4**
4	Protibiae exterior with two lobes (one on the distal end and one closer to the proximal end)	**5**
–	Protibiae exterior simple (lacking lobes) or with a singular lobe that can be present partially on the distal portion only or fully spanning the shaft	**6**
5	Gonapophyses VIII average in length, with a majority of their length under the terminal abdominal segment, only the tips project from the apex; tegmina venation has the radius and the media diverging immediately, not running parallel for any portion of their lengths; tegmina venation has a distinct radius to media crossvein	***Vaabonbonphyllium* gen. nov.**
–	Gonapophyses VIII long, with ca. half of their length projecting from under the terminal abdominal segment; tegmina venation has the radius running alongside the media until the radial sector arcs away; tegmina venation, no radius to media crossvein present	***Rakaphyllium* gen. nov.**
6	Pro-, meso-, and metatibiae exterior with lobes; and tegmina with media and cubitus veins running side by side, touching throughout most of their lengths	***Pulchriphyllium* Griffini, 1898**
–	Pro-, meso-, and meatibiae simple, lacking exterior lobes (but if exterior lobes are present they are usually only partially present only on the distal portion or very rarely fully spanning the shaft (in which case the tegmina media and cubitus veins are distinctly separated with several vein width distances between them throughout the length, not touching))	**7**
7	Prescutum spination along the sagittal plane with the middle or posterior spine the most prominent (therefore not equal in size the other spines)	**8**
–	Prescutum sagittal plane either with: the spine on the anterior rim most prominent, an anterior rim followed by granulation, or with spination of equal size from the anterior to the posterior (no single spine notably more prominent than the others)	**9**
8	Posterior most spine of the prescutum is the most prominent; gonapophyses VIII which are long, reaching to the apex of the anal abdominal segment; third antennomere with stridulatory file	***Pseudomicrophyllium* Cumming, 2017**
–	Middle most spine along the prescutum is the largest spine and the posterior most spine is highly reduced; gonapophyses VIII are notably short, only slightly protruding from under the subgenital plate; third antennomere lacking stridulatory file	***Microphyllium* Zompro, 2001a**
9	Terminal antennomere as long as the preceding three to five segments combined	***Comptaphyllium* Cumming et al., 2019**
–	Terminal antennomere as long as the preceding one or two segments combined	**10**
10	Tegmina with media and cubitus veins running side by side, touching throughout the majority of their lengths or fused throughout most of their length	**11**
–	Tegmina with media and cubitus veins with significant spacing between them (several vein widths away) not touching	**12**
11	Coxae ventrally are sky blue in color; prescutum anterior rim strongly protruding and distinctly angled posteriorly; tegmina radial vein (R) diverges steadily from media (M) for the full length, therefore the split of the radial sector (Rs) is not a significant bend	***Chitoniscus* Stål, 1875**
–	Coxae ventral color is the same color as the legs and thorax; prescutum anterior rim and spine not strongly protruding posteriorly, only slightly raised and vertical; tegmina radial vein (R) runs parallel with media (M) until the split of the radial sector (Rs), at which point the radial sector (Rs) bends away distinctly	***Trolicaphyllium* Cumming et al. 2021**
12	Third antennomere with the proximal end broad and often slightly recurved, making the segment a similar width throughout, or broader on the proximal end; fourth antennomere short and disk-like at least 3× wider than long and notably shorter than any of the following three segments, or rarely a similar length to the following segment, but still at least 2× as wide as long	***Cryptophyllium* Cumming et al., 2021**
–	Third antennomere narrowest on the proximal end, broadening to the distal end; fourth antennomere typically as long as wide and of a similar length to each of the following three segments length, not notably shorter	***Phyllium* Illiger, 1798**

### ﻿Key to males

**Table:** 

1	Posteriormedial tubercle of the head capsule split into two lobes	**2**
–	Posteriormedial tubercle of the head capsule singular, not split into two lobes	**3**
2	The profemoral interior lobes are angular; mesofemoral interior lobes are heavily weighted towards the distal end and are marked by distinct serrate teeth on the distal half; alae, the media posterior fuses to the media anterior and then they run fused to the wing margin without fusing with other veins	***Nanophyllium* Redtenbacher, 1906**
–	The profemoral interior lobes are rounded without a sharp angle; mesofemoral interior lobes have a large rounded triangular lobe, reaching from end to end and lacking prominent spination; alae, the media anterior and the media posterior do not fuse, instead they both run to the wing margin, and the cubitus after splitting from the first anterior anal fuses with the media posterior near the wing margin and they run fused to the wing apex	***Acentetaphyllium* gen. nov.**
3	Alae, radius vein is simple, does not split	***Rakaphyllium* gen. nov.**
–	Alae, radius vein is bifurcate, creating the radial sector (Rs) and first radius (R1)	**4**
4	Small (< 30.0 mm in length); protibiae lacking an interior lobe; restricted to the Philippines	**5**
–	Medium to large (35.0 mm to > 80.0 mm); protibiae almost always with a half to fully developed interior lobe, or rarely highly reduced to a sliver on the proximal half only.	**6**
5	Antennae short (only ca. the length of the outstretched front legs), with bead-like antennomeres that are no more than 2× longer than they are wide; profemoral interior lobes which are narrow and marked with three distinct teeth	***Microphyllium* Zompro, 2001a**
–	Antennae notably longer than the outstretched front legs, with antennomeres 4–5× longer than wide; profemoral interior lobes reduced to only a single anterior spine	***Pseudomicrophyllium* Cumming, 2017**
6	Prescutum stout, ca. 2× wider than long	**7**
–	Prescutum as long as wide or notably longer than wide	**8**
7	Ocelli absent; alae, media anterior (MA) runs unfused to the wing margin and media posterior (MP) fades without fusing or reaching the wing margin	***Chitoniscus* Stål, 1875**
–	Ocelli well-developed; alae, media anterior (MA) and media posterior (MP) veins fuse with the cubitus (Cu) at different locations along the cubitus and run fused to the wing margin	***Trolicaphyllium* Cumming et al. 2021**
8	Vomer with two apical hooks	***Cryptophyllium* Cumming et al., 2021**
–	Vomer with a single apical hook	**9**
9	Profemora exterior with two lobes, one near the middle of the length and one on the distal end	***Vaabonbonphyllium* gen. nov.**
–	Profemora exterior either with one lobe or simple, lacking lobes	**10**
10	Alae radial sector, media anterior, and media posterior veins fusing to the cubitus at different locations along the vein and running together to the wing margin; metafemora exterior with a prominent lobe spanning the full length of the shaft	***Pulchriphyllium* Griffini, 1898**
–	Alae radial sector, media anterior, and media posterior not fusing with the cubitus; metafemora exterior simple, lacking a lobe	**11**
11	Tegmina media vein splits into the anterior media vein (MA) and posterior media vein (MP) very early on, immediately or at most 1/4 of the way through the wing length and they run unbranched and subparallel through the wing length; protibial interior lobe not reaching from end to end of the shaft, only restricted to the proximal 1⁄2 to 2⁄3 but never more; a head capsule with clearly defined nodes arranged in evenly spaced patterns	***Comptaphyllium* Cumming et al., 2019**
–	Tegmina media vein running unbranched for the first 1⁄3 to 2⁄5 of the wing length, and then branching with either a single short media posterior running to the wing margin or two short media posteriors branching from the notably longer media anterior and running to the margin; protibial interior lobe variable, either fully spanning the full length or only 1⁄2 of the length; head capsule at most with random granulation but frequently bare	**12**
12	Abdominal shape rectangular, with segments V and VI with fully parallel-sided margins (segments IV and VII with only half parallel-sided and the remainder curved)	***Walaphyllium* Cumming et al., 2020b**
–	Abdominal shape variable, either spade-shaped (with the margins of V parallel or strongly converging and segment VI strongly converging), ovular (with margins expanding and then contracting, no segments parallel-sided), thin and slender with converging margins, bell-shaped (with margins expanding until segment VI then strongly converging) or boxy with only segment V parallel-sided (segments IV and VI only partially parallel-sided, the remainder rounded)	***Phyllium* Illiger, 1798**

## ﻿Conclusions

*Melanesia* has been recovered as the likely origin of all extant phylliids with a possible origin around ~ 50 million years ago (MYA) during the Eocene (Bank et al. 2021). Given this recovered history it logically follows that as this region is home to older lineages which have had significantly more time for morphological diversification, this region has more distinct phylliid clades (which based upon their prominent morphological distinctiveness are treated by taxonomists as genera). In comparison, the regions which were more recently colonized (~ 20 to 25 MYA) such as mainland Asia, Sundaland, and the Philippines have a high diversity of younger lineages and not as many lineages treated as genera due to their more recent diversification and significant morphological similarities (Bank et al. 2021). As the phylliids of *Melanesia* have been reviewed in the past few years, through detailed morphological examination of eggs, freshly hatched nymphs, and fine details of adults, coupled with molecular analyses, the significant morphological diversity of this region is now being described with more clarity.

This region is, however, severely under-collected as many of the species of *Melanesia* are only known from type specimens or only a few representatives of each species. Likely the species diversity of *Melanesia* is also notably diverse, but due to this limited sampling species ranges and diversity are only beginning to be unraveled. Hopefully future research efforts in *Melanesia* will recover representatives of these herein described genera which have yet to be sequenced to include them in molecular phylogenies to clarify the higher-level taxonomic classifications of the leaf insects.

## Supplementary Material

XML Treatment for Rakaphyllium

XML Treatment for Rakaphyllium schultzei

XML Treatment for Rakaphyllium exsectum

XML Treatment for Vaabonbonphyllium

XML Treatment for Vaabonbonphyllium groesseri

XML Treatment for Vaabonbonphyllium rafidahae

XML Treatment for Acentetaphyllium

## Supplementary materials

Supplementary material 1 Material examined Data type specimen data Explanation note Material examined (specimen data and deposition/ observational records) for localities used within the distribution map (Fig. 5) and to accompany the discussion of each species. https://binary.pensoft.net/file/710975This dataset is made available under the Open Database License (http://opendatacommons.org/licenses/odbl/1.0/). The Open Database License (ODbL) is a license agreement intended to allow users to freely share, modify, and use this Dataset while maintaining this same freedom for others, provided that the original source and author(s) are credited.Royce Cumming, Stéphane Le Tirant

Supplementary material 2 List of species/specimens included within the phylogenetic analyses of this study (molecular data portion) Data type molecular sequence data accession numbers Explanation note List of species/specimens included within the phylogenetic analyses of this study (molecular data portion). Taxonomic information, specimen collection locality, sample ID, and collection deposition are provided. GenBank accession numbers are listed for each sequence used. https://binary.pensoft.net/file/710976This dataset is made available under the Open Database License (http://opendatacommons.org/licenses/odbl/1.0/). The Open Database License (ODbL) is a license agreement intended to allow users to freely share, modify, and use this Dataset while maintaining this same freedom for others, provided that the original source and author(s) are credited.Royce Cumming, Stéphane Le Tirant

Supplementary material 3 Nexus file containing the molecular sequences and morphological data Data type molecular and morphological sequence data Explanation note Nexus file containing the molecular sequences and morphological data utilized within the phylogenetic analyses. https://binary.pensoft.net/file/710977This dataset is made available under the Open Database License (http://opendatacommons.org/licenses/odbl/1.0/). The Open Database License (ODbL) is a license agreement intended to allow users to freely share, modify, and use this Dataset while maintaining this same freedom for others, provided that the original source and author(s) are credited.Royce Cumming, Stéphane Le Tirant

Supplementary material 4 List of adult morphological characters and their states Data type morphological features and their available states Explanation note List of adult morphological characters and their states used to code the morphological matrices. https://binary.pensoft.net/file/710978This dataset is made available under the Open Database License (http://opendatacommons.org/licenses/odbl/1.0/). The Open Database License (ODbL) is a license agreement intended to allow users to freely share, modify, and use this Dataset while maintaining this same freedom for others, provided that the original source and author(s) are credited.Royce Cumming, Stéphane Le Tirant

Supplementary material 5 List of species and matrix of morphological characters for adults of outgroup phasmid and representatives from all Phylliidae genera for phylogenetic analyses Data type morphological matrix data Explanation note List of species and matrix of morphological characters for adults of outgroup phasmid and representatives from all Phylliidae genera for phylogenetic analyses. https://binary.pensoft.net/file/710979This dataset is made available under the Open Database License (http://opendatacommons.org/licenses/odbl/1.0/). The Open Database License (ODbL) is a license agreement intended to allow users to freely share, modify, and use this Dataset while maintaining this same freedom for others, provided that the original source and author(s) are credited.Royce Cumming, Stéphane Le Tirant

Supplementary material 6 Mr. Bayes run logs for the male and female matrices Data type phylogenetic analyses run logs with settings Explanation note Mr. Bayes run logs for the male and female matrices. https://binary.pensoft.net/file/710980This dataset is made available under the Open Database License (http://opendatacommons.org/licenses/odbl/1.0/). The Open Database License (ODbL) is a license agreement intended to allow users to freely share, modify, and use this Dataset while maintaining this same freedom for others, provided that the original source and author(s) are credited.Royce Cumming, Stéphane Le Tirant

Supplementary material 7 Results of parsimony analyses on morphological data matrices Data type supplementary phylogenetic results Explanation note Results of parsimony analyses on morphological data matrices https://binary.pensoft.net/file/710981This dataset is made available under the Open Database License (http://opendatacommons.org/licenses/odbl/1.0/). The Open Database License (ODbL) is a license agreement intended to allow users to freely share, modify, and use this Dataset while maintaining this same freedom for others, provided that the original source and author(s) are credited.Royce Cumming, Stéphane Le Tirant
