# Supplementary material for: ﻿Three new genera and one new species of leaf insect from Melanesia (Phasmatodea, Phylliidae)
Source: Zookeys. 2022 Jul 5;1110:151–200. doi: 10.3897/zookeys.1110.80808 (PMC9848868; doi:10.3897/zookeys.1110.80808)
Supplement: Supplementary material 4 — List of adult morphological characters and their states [file zookeys-1110-151_article-80808__-s004.docx]

**Supplementary File 4.**

List of adult morphological characters and their states used to code the matrices.

Separated by sex.

**Female**

1. **Female; number of antennae segments**

(0): 9 segments.

(1): 10 segments.

(2): 11+ segments.

1. **Female; terminal antennomere (length relative to other segments)**

(0): Terminal antennomere as long as the previous one to two segments lengths combined.

(1): Terminal antennomere as long as the previous three to five segments lengths combined.

1. **Female; third antennomere (stridulatory file and ridge)**

(0): Stridulatory file and ridge absent, at most a few setae.

(1): Stridulatory file and ridge present.

1. **Female; stridulatory file of the third antennomere (# of teeth)**

(0): No teeth.

(1): 15 to 20 teeth.

(2): 20 to 30 teeth.

(3): 31 to 40 teeth.

(4): 41 to 50 teeth.

(5): 51 to 60 teeth.

1. **Female; ocelli**

(0): Absent.

(1): Distinctly developed.

1. **Posteriormedial tubercle of the head capsule**

(0): Singularly pointed.

(1): Bilobed, split into two points.

1. **Female; prescutum anterior rim (angle)**

(0): Prescutum anterior rim and spine prominent and strongly angled posteriorly.

(1): Prescutum anterior rim and/or spine prominent but not protruding posteriorly, vertical instead.

(2): Prescutum lacking a prominently protruding anterior rim, can be basically devoid of a raised rim or only a weakly formed anterior rim is present lacking a spine.

1. **Female; prescutum sagittal spine(s)**

(0): No spines present (at most just a weak anterior rim).

(1): Anterior spine the most prominent (can be smaller spines following).

(2): Middlemost spine the largest.

(3): Posteriormost spine the largest.

(4): Multiple spines from the anterior to the posterior and all are the same size.

1. **Female; prescutum sagittal crest (arrangement of nodes/tubercles)**

(0): Distinct nodes/weakly formed tubercles arranged throughout the prescutum surface, not only along the sagittal plane.

(1): Most prominent nodes/granulation//tubercles arranged along the sagittal plane, prescutum surface smooth or at most slightly lumpy.

1. **Female; prescutum sagittal crest tubercles**

(0): Anterior most tubercle the most prominent, all others smaller (whether or not they are arranged along the sagittal plane or the full prescutum).

(1): All tubercles along the sagittal crest prominent and the same size.

(2): Posterior most prominent and other tubercles variable in size or middle tubercle most prominent, not the anterior tubercle.

(3): Sagittal crest lacks distinct tubercles, usually just granulation or a weak anterior rim and granulation.

1. **Female; prosternum spine**

(0): Absent, prosternum with granulation or smooth.

(1): Notable projection present near the center of the prosternum, can be large and notably raised or a prominent tubercle.

1. **Female; mesopleurae (general shape)**

(0): Narrow on the anterior margin and beginning to diverge gradually throughout the full length.

(1): Narrow/parallel for approximately the anterior third before beginning to diverge.

(2): Narrow/parallel for approximately the anterior half before beginning to diverge.

(3): Anterior distinctly wider than the prescutum anterior width and diverging throughout their lengths.

(4): Narrow on the anterior half, then strongly diverging and armored with a prominent spine in the center notably larger than any tubercles present.

(5): Mesothorax tubular, no laterally projecting mesopleurae.

1. **Female; prescutum length to width ratio**

(0): Distinctly longer than wide.

(1): Approximately as long as greatest width.

(2): Widest point distinctly wider than long.

1. **Female; tegmina (length)**

(0): Rudimentary, at most only reaching the anterior margin of abdominal segment II.

(1): Well-developed but stunted, only reaching onto abdominal segments V-VI.

(2): Well-developed and long, reaching onto segments VII to X.

(3): Absent.

(4): Rudimentary, only small flaps without distinct venation which do not reach the abdomen.

(5): Rudimentary, only reaching onto abdominal segment III.

(6): Rudimentary, not reaching abdomen, but with distinct venation present.

1. **Female; tegmina (first radius)**

(0): First radial (R1) diverges from the radius early on, less than half of the way between the wing base and the radius to media cross vein (R–M)/the bend in the radial sector.

(1): First radial (R1) diverges from the radius ranging from ca. 1/2 of the way between the wing base and the radius to media cross vein (R–M)/the bend in the radial sector to close to the radius to media cross vein (R–M)/the bend in the radial sector.

(2): First radial (R1) diverges from the radius after the radius to media cross vein (R–M)/the bend in the radial sector.

(3): Tegmina heavily sclerotized and the radius vein is simple, not bifurcate.

(4): Radial sector(s) diverge from the radius ca. ½ through the tegmina length.

1. **Female; tegmina (radius and media)**

(0): Radius and the media diverging immediately and throughout their lengths, not running parallel for any portion.

(1): Radius straight running alongside the media (either touching or parallel with it no more than three vein widths away) until the radial sector arcs away.

(2): Radius and media arc together side by side and then the radius bends perpendicular to the media and runs through the center of the tegmina.

(3): Radius and media diverge immediately but run subparallel/parallel throughout their full lengths multiple vein widths apart.

1. **Female; tegmina (media and cubitus)**

(0): Media and cubitus veins run straight fused/touching throughout most of their length.

(1): Media and cubitus run straight with at least a vein width between them, not touching, but often a more significant gap of several vein widths is present.

(2): Media and cubitus veins arc along the rounded edge of the tegmina with greater than 1 vin width between them.

1. **Female; tegmina (cubitus)**

(0): Tegmina venation with the posterior cubitus split into an anterior cubitus (CuA), first posterior cubitus (CuP1), and second posterior cubitus (CuP2).

(1): Tegmina cubitus bifurcate (into an anterior cubitus (CuA) and posterior cubitus (CuP1) only).

(2): Tegmina cubitus venation simple (unsplit).

1. **Female; alae (length)**

(0): Alae rudimentary, only a nub.

(1): Alae weakly developed; venation present but alae less than 1/2 of the tegmina length (longer than 5.0 mm).

(2): Alae fully developed, greater than 1/2 the length of the tegmina

(3): Absent.

(4): Tegmina and alae the same length, both rudimentary, only reaching to abdominal segment III.

1. **Female; alae (radius split)**

(0): Radial split into first radial and radial sector happens near the proximal third of the wing.

(1): Radial split into first radial and radial sector happens near the middle of the alae length.

(2): Radius simple, does not split.

1. **Female; ventral meso- and meta- coxae coloration**

(0): Colored differently from the general base coloration.

(1): Colored the same to the surrounding tissue.

1. **Female; metasternum warning display coloration**

(0): Distinct warning display coloration present on the membrane between the metasternum and abdominal segment II.

(1): No warning coloration present, membrane color identical to surrounding coloration.

1. **Female; protibiae, exterior lobe**

(0): Simple, lacking a lobe.

(1): Lobe well-developed, fully spanning the length.

(2): Single lobe partially developed on the proximal half, occupying less than half of the length.

(3): Two small lobes present.

(4): Single small lobe on the distal end only.

1. **Female; protibiae, interior lobe**

(0): Absent.

(1): Present, but not fully spanning the protibial shaft.

(2): Present, fully spanning the protibial shaft.

1. **Female; profemoral shaft shape**

(0): Profemora shaft basally curved.

(1): Profemora shaft straight.

1. **Female; profemoral exterior lobe (general shape)**

(0): Arcing smoothly from end to end without a sharp angle and thinner than the profemoral interior lobe.

(1): Arcing from end to end with/without a slight obtuse angle (can be smoothly arcing end to end or with a gently rounded obtuse bend in the middle when the lobe is notably wide) and wider than the profemoral interior lobe.

(2): 1.5 times wider than the interior lobe and with a rounded right angle.

(3): Arcing from end to end and the same width as the profemoral interior lobe.

(4): Approximately 2.0 times wider than the interior lobe and with a right angle or acute angle.

(5): Approximately 1.5 times wider than the interior lobe and with a recurved acute angle.

1. **Female; mesotibiae, exterior lobe**

(0): Simple, lacking a lobe.

(1): Lobe well-developed, fully spanning the length.

(2): Lobe partially developed, occupying ca. half of the length.

(3): Small lobe present on the distal tip only.

(4): Small lobe in the center of the shaft.

(5): Small lobe present on the proximal portion only.

1. **Female; mesofemoral interior lobe**

(0): As wide as the mesofemoral shaft and maked with a serrate distal margin.

(1): Notably wider than the mesofemoral shaft and maked with a serrate distal margin.

(2): Notably wider than the mesofemoral shaft and maked with a smooth distal margin which can be held alongside the body to create a smooth continuous margin.

(3): No well-developed lobe, mesofemoral shaft only, most/all of the length is without a lobe.

1. **Female; metatibiae, exterior lobe**

(0): Simple, lacking a lobe.

(1): Lobe well-developed, fully spanning the length.

(2): Lobe partially developed, occupying ca. half of the length.

(3): Small lobe present on the distal tip only.

(4): Small lobe in the center of the shaft.

1. **Female; abdomen flattened**

(0): Abdomen sternite and tergites projecting laterally into leaf-like expansions.

(1): Abdomen lacking lateral projections, instead tubular.

1. **Female; subgenital plate (length ratio)**

(0): Plate short, length from apex to posterior margin of abdominal sternite VIII less than length from the posterior margin of abdominal sternite VIII and the anterior margin of abdominal sternite VIII.

(1): Plate medium length, length from apex to posterior margin of abdominal sternite VIII equal to the length from the posterior margin of abdominal sternite VIII and the anterior margin of abdominal sternite VIII.

(2): Plate long, length from apex to posterior margin of abdominal sternite VIII greater than length from the posterior margin of abdominal sternite VIII and the anterior margin of abdominal sternite VIII.

1. **Female; gonapophyses VIII (length)**

(0): Moderate in length, tips reaching or very slightly exceeding the apex of the abdomen with a majority of their length unexposed.

(1): Exceedingly long, approximately half of their length projecting from under the apex of the abdomen.

(2): Notably short, extending no more than halfway under the terminal abdominal segment.

1. **Female; gonapophyses VIII (width)**

(0): Slender, ca. as wide as the cerci width.

(1): Moderately wide, 1.5 to 2.0 times as wide as the cerci width.

(2): Broad, greater than 2.0 times wider than the cerci.

**Male**

1. **Male; ocelli**

(0): Absent, can be entirely flat and absent or with a slightly raised area where ocelli should be, but no ocelli markings present.

(1): Moderately developed.

(2): Distinctly developed.

1. **Male; number of antenna segments**

(0): 20 to 30 segments.

(1): 40+ segments.

1. **Male; overall antennae length**

(0): Antennae notably shorter than outstretched front legs.

(1): Antennae notably longer than outstretched front legs.

(2): Antennae similar in length to outstretched front legs.

1. **Male; antennomere length vs. width**

(0): Antenna segments short and bead-like, most segments are two times longer than wide, and only a few of the longest segments are ca. three times longer than wide (but not longer) when viewed dorsally.

(1): Antenna segments long and thin, a majority of segments are at least three to five times longer than wide when viewed dorsally.

1. **Posteriormedial tubercle of the head capsule**

(0): Singularly pointed.

(1): Split into two points.

1. **Male; pronotum width/length ratio**

(0): Length similar to greatest width.

(1): Longer than greatest width.

(2): Greatest width greater than length.

1. **Male; mesopleurae (general shape)**

(0): Narrow on the anterior margin and beginning to diverge immediately.

(1): Narrowly beginning and parallel for approximately the anterior third before beginning to diverge.

(2): Narrowly beginning and parallel for approximately the anterior half before beginning to diverge.

(3): Anterior distinctly wider than the prescutum anterior width and diverging throughout their lengths.

(4): Narrow on the anterior half, then strongly diverging and armored with a prominent spine in the center notably larger than any tubercles present.

(5): Distinctly wider on the anterior, but parallel sided for the anterior half, and then diverging.

(6): Mesothorax tubular, no laterally projecting mesopleurae.

1. **Male; prescutum sagittal spine(s)**

(0): No spines present (at most just a weak anterior rim and a few granules along the sagittal plane but none prominent).

(1): Anterior spine the most prominent (can be smaller spines following).

(2): Middlemost spine the most prominent.

(3): Posteriormost spine the most prominent.

(4): Multiple spines from the anterior to the posterior and all are the same size.

(5): Spines present but not along the sagittal plane, either arranged throughout in no discernible pattern or only laterally.

1. **Male; prescutum length to width ratio**

(0): Distinctly longer than wide.

(1): Approximately as long as widest point.

(2): Distinctly wider than long.

1. **Male; tegmina length**

(0): Short, length never exceeding the posterior of the metathorax.

(1): Moderate in length, reaching onto abdominal segment II or III.

(2): Long, length exceeding abdominal segment IV or V.

(3): Absent, no tegmina.

(4): Rudimentary, only small flaps without distinct venation which do not reach the abdomen.

1. **Male; tegmina (radial splits)**

(0): A singular first radial (R1) splits from the radial sector (Rs).

(1): Multiple radial veins (R1, R2, etc.) split from the radial sector (Rs).

(2): Tegmina heavily sclerotized and radius appears to be simple, no distinct splitting.

1. **Male; tegmina (media splits)**

(0): A singular media posterior (MP) originates from near the tegmina base and runs slowly diverging from the media anterior (MA).

(1): A singular media posterior splits from somewhere along the tegmina length (typically near the middle of the tegmina length).

(2): Multiple media posteriors (MP1, MP2, etc.) split from the media anterior (MA) throughout the tegmina length.

(3): Tegmina heavily sclerotized and media appears to be simple, no distinct splitting.

1. **Male; alae length**

(0): Absent, no alae.

(1): Fully developed alae reaching to apex of the abdomen.

(2): Fully developed alae which reach onto the abdomen but do not reach to the apex (instead only reaching segments IV through VII).

(3): Alae rudimentary, barely reaching past the posterior of the metanotum.

1. **Male; alae radius vein**

(0): Singularly veined, not split.

(1): Bifurcate split into R1 and Rs on the proximal half of the alae.

(2): Bifurcate split into R1 and Rs on the distal half of the alae.

(3): Bifurcate split into R1 and Rs happens in the middle of the alae.

1. **Male; alae media vein**

(0): Media posterior fuses with the media anterior and these fuse with the radial sector before reaching wing apex creating MA+MP+Rs.

(1): Media posterior fuses with the media anterior and this runs to the wing apex (or fades slightly before reaching it) as MA+MP.

(2): Media anterior runs alone to the alae apex, media posterior fuses with cubitus and they run to apex as MP+Cu.

(3): Media posterior and media anterior fuse independently to the cubitus and run to the apex as MA+MP+Cu.

(4): Media posterior fades before connecting to another vein, media anterior fuses with radial sector and these run to apex as MA+Rs.

(5): Both the media anterior and the media posterior fade independently before fusing with other veins or reaching the alae apex.

(6): Media anterior vein reaches the alae apex without fusing with other veins or fading, media posterior fades before fusing with other veins or reaching the apex.

(7): First radial, radial sector, media anterior, and media posterior fuse with the cubitus independently and the Cu+R1+Rs+MA+MP runs to the apex.

(8): The media anterior and the media posterior run independently fully to the alae apex without fusing to others and without fading.

1. **Male; alae cubitus vein**

(0): Runs independently to the alae apex.

(1): Media posterior fuses alone to the cubitus and they run to the apex as MP+Cu.

(2): Media anterior and media posterior fuse to the cubitus independently and the Cu+MA+MP runs to the apex.

(3): First radial, radial sector, media anterior, and media posterior fuse with the cubitus independently and the Cu+R1+Rs+MA+MP runs to the apex.

(4): Cubitus and first anterior anal are fused and run as such to the alae apex as Cu+AA1.

1. **Male; protibiae, exterior lobe**

(0): Simple, lacking a lobe.

(1): Lobe well-developed, fully spanning the length.

(2): Lobe partially developed, occupying half or less of the length.

(3): Two small lobes present.

(4): Single small lobe in the center of the shaft.

1. **Male; protibiae, interior lobe**

(0): Absent.

(1): Present, but not fully spanning the protibial shaft.

(2): Present, fully spanning the protibial shaft.

1. **Male; profemoral exterior lobe (general shape)**

(0): Arcing smoothly from end to end without a sharp angle and thinner than the profemoral interior lobe.

(1): Arcing from end to end and the same width as the profemoral interior lobe (can be smoothly arcing or with a slight obtuse angle).

(2): Arcing smoothly from end to end and wider than the profemoral interior lobe.

(3): Notably wider than the interior lobe and with a right angle or acute angle.

(4): Small (only occupying the central portion of the shaft), the same width as the interior lobe, and right or acutely angled.

(5): Simple, lobe lacking, only shaft present.

1. **Male; profemoral interior lobe**

(0): Lobe absent, profemoral shaft unadorned.

(1): Lobe present but very thin (no wider than the width of the profemoral shaft) and marked with three small teeth.

(2): Only a small spur is present on the distal end, the majority lacks a lobe.

(3): Lobe distinctly developed, at least two times wider than the profemoral shaft width.

1. **Male; mesotibiae, exterior lobe**

(0): Simple, lacking a lobe.

(1): Lobe well-developed, fully spanning the length.

(2): Lobe partially developed, occupying ca. half of the length.

(3): Small lobe present on the distal tip only.

(4): Two small lobes, one near the center of the shaft and one on the distal tip.

(5): Small lobe present in the center only.

1. **Male; metatibiae, exterior lobe**

(0): Simple, lacking a lobe.

(1): Lobe well-developed, fully spanning the length.

(2): Lobe partially developed, occupying ca. half of the length.

(3): Small lobe present on the distal tip only.

(4): Small lobe in the center of the shaft.

1. **Male; general abdomen**

(0): Abdomen sternite and tergites projecting laterally into leaf like expansions.

(1): Abdomen lacking lateral projections, instead tubular.

1. **Male; vomer (number of apical hooks)**

(0): Singularly hooked.

(1): Doubly hooked, with a primary apical hook and an axillary smaller hook adjacent.

1. **Male; vomer (general shape)**

(0): Distinctly wider than long.

(1): Distinctly longer than wide.

(2): Approximately as long as wide.
