# Supplementary material for: ﻿Three new genera and one new species of leaf insect from Melanesia (Phasmatodea, Phylliidae)
Source: Zookeys. 2022 Jul 5;1110:151–200. doi: 10.3897/zookeys.1110.80808 (PMC9848868; doi:10.3897/zookeys.1110.80808)
Supplement: Supplementary material 7 — Results of parsimony analyses on morphological data matrices [file zookeys-1110-151_article-80808__-s007.docx]

Supplementary File 7.

Results of parsimony analyses on morphological data matrices.


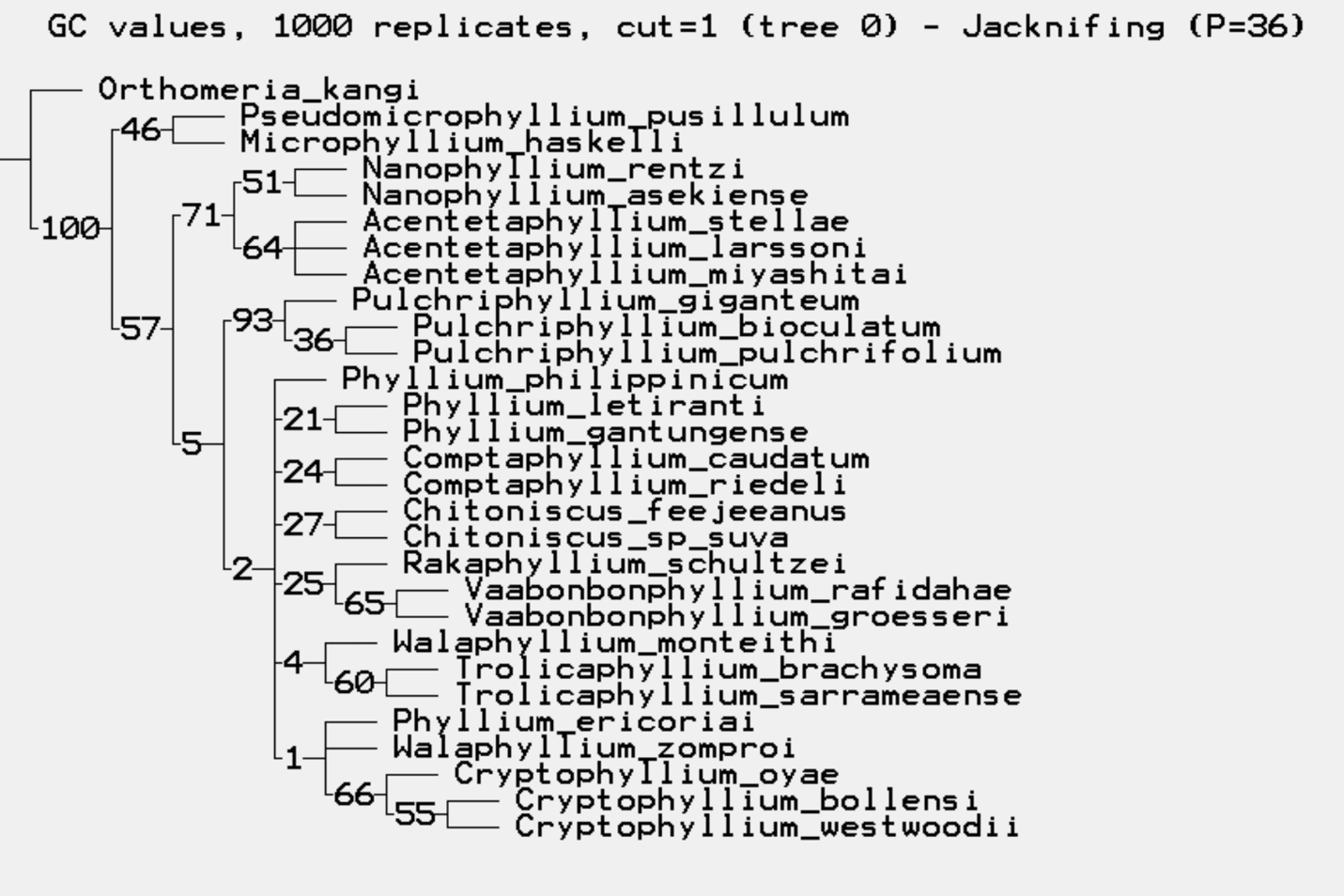


Figure 1. Male results showing support values calculated by jackknifing.


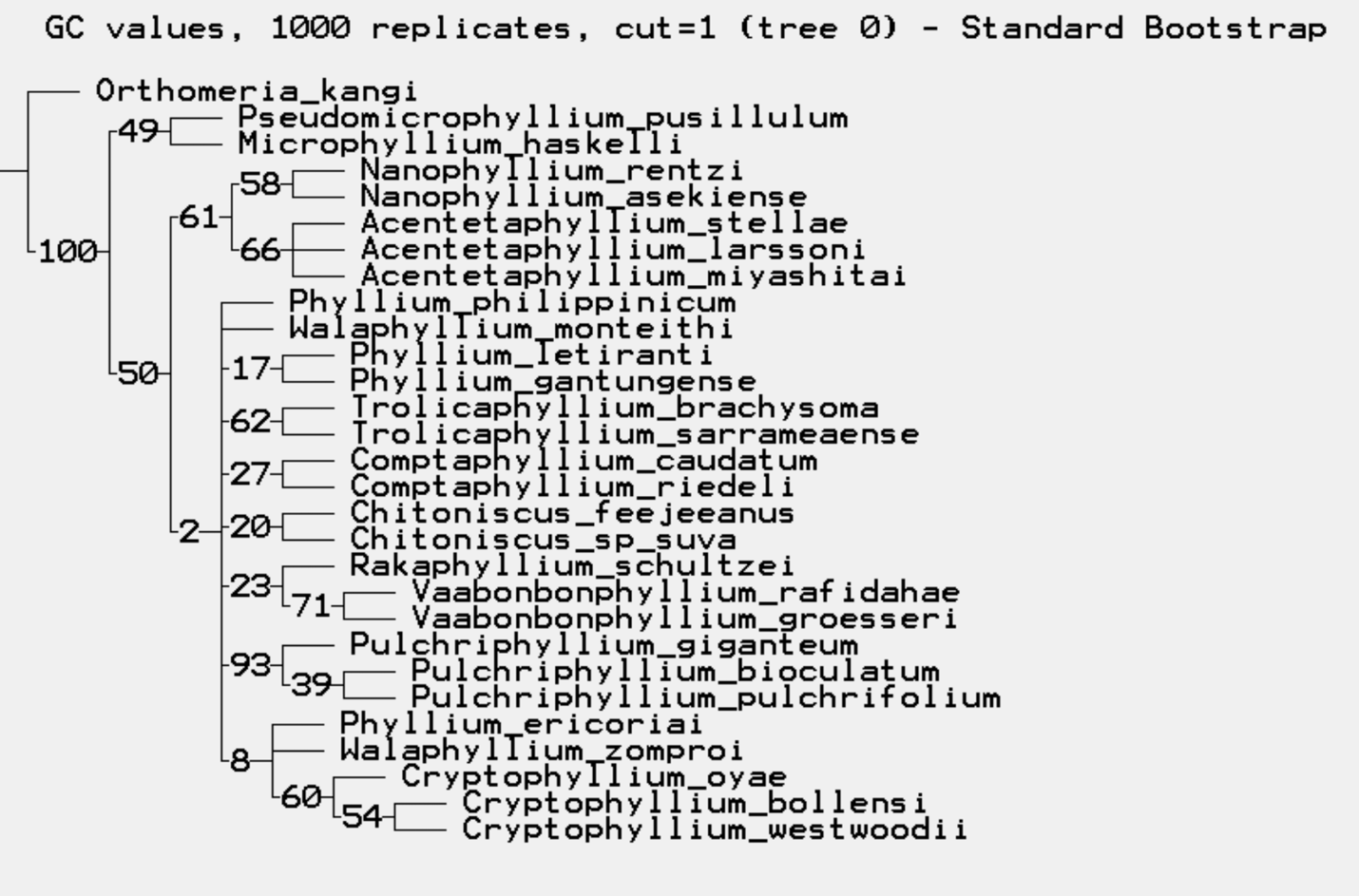


Figure 2. Male results showing support values calculated by the standard bootstrap.


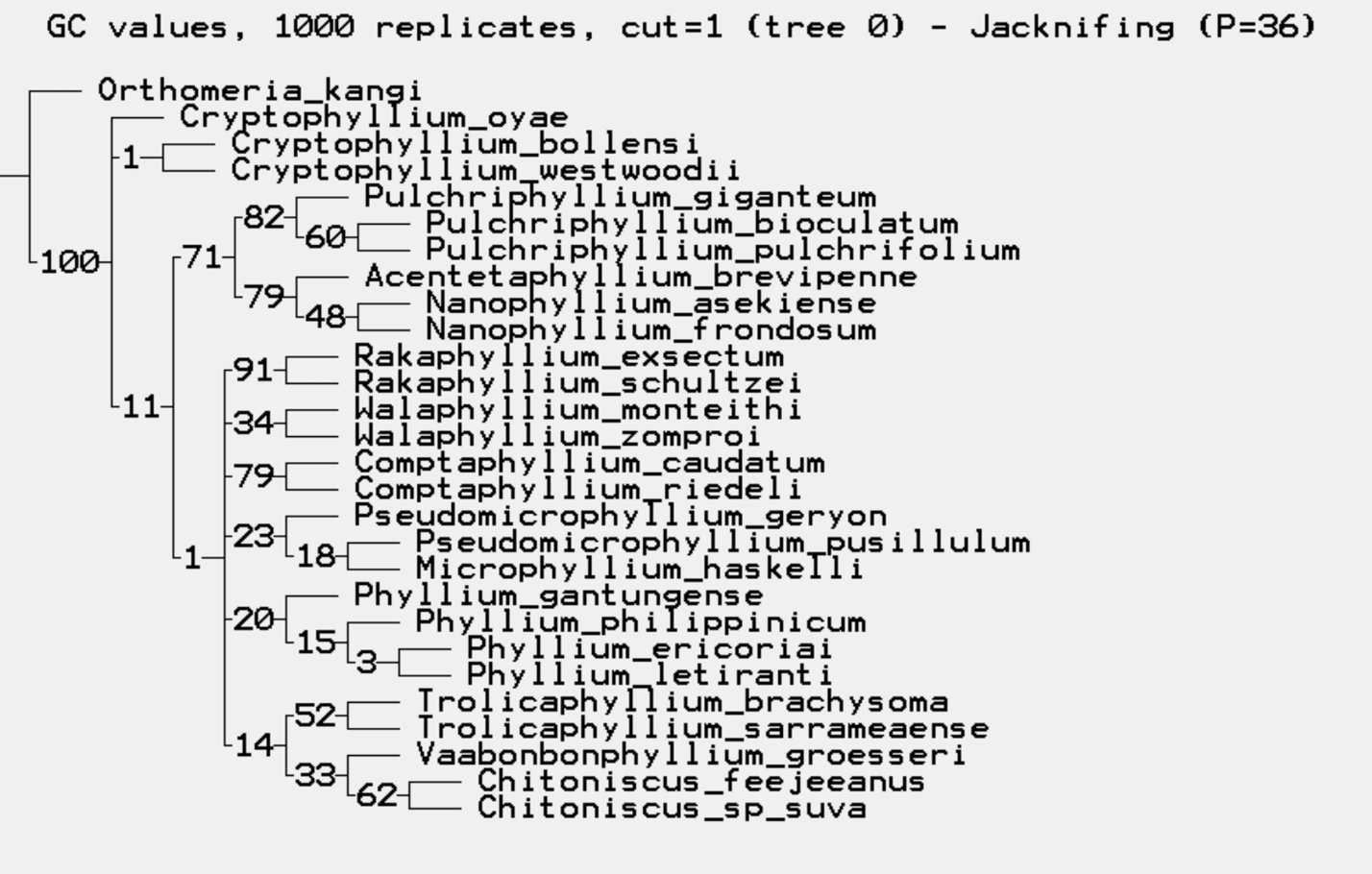


Figure 3. Female results showing support values calculated by jackknifing.


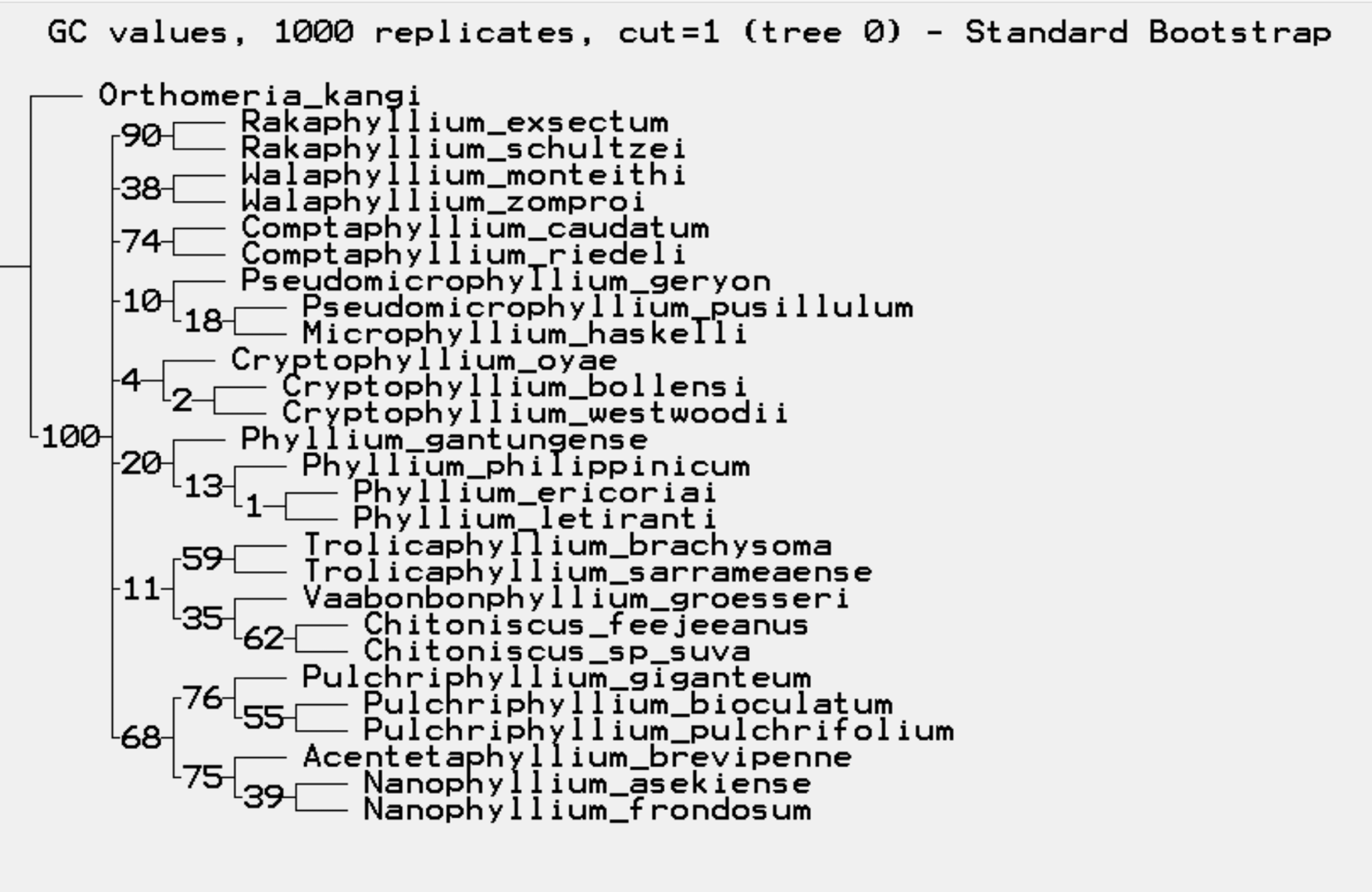


Figure 4. Female results showing support values calculated by the standard bootstrap.
